# Evaluating Laryngeal Stability in Drug-Induced Parkinsonism Using Hilbert–Huang Transform and Chaos Dynamics: A Multi-Vowel Nonlinear Acoustic Framework for Differential Diagnosis

**DOI:** 10.3390/diagnostics16193187

**Published:** 2026-09-30

**Authors:** Andrew An-Zhe Lee, Chun-Hung Lee

**Affiliations:** 1 School of Medicine, College of Medicine, National Yang Ming Chiao Tung University, Taipei 112304, Taiwan; 2 Department of General Psychiatry Taoyuan Psychiatric Center, Ministry of Health and Welfare, Taoyuan 330, Taiwan; 3 Department of Education, National Taiwan Normal University, Taipei 11677, Taiwan; 4 Department of Information Engineering, I-Shou University, Kaohsiung 84001, Taiwan

**Keywords:** drug-induced parkinsonism, extrapyramidal syndrome, Parkinsonism, laryngeal nonlinear acoustic, Hilbert–Huang Transform, entropy, correlation dimension, Recurrence Quantification Analysis, dyskinesia

## Abstract

**Background/Objectives**: Drug-induced parkinsonism (DIP) is a common extrapyramidal adverse effect of dopamine-receptor blocking agents, yet interpretable biomarkers of its acoustics remain limited. This study investigated whether nonlinear dynamics could provide physiologically interpretable markers of DIP and clarify how these abnormalities vary across vowels and extrapyramidal symptoms. **Methods**: Sustained phonations of five vowels (/a/, /i/, /u/, /e/, /o/) were analyzed in 82 patients with DIP and 30 healthy controls. Hilbert–Huang Transform (HHT) features were integrated along with Sample Entropy, Correlation Dimension, Largest Lyapunov Exponent, and Recurrence Quantification Analysis (RQA). Associations with Drug-Induced Extrapyramidal Symptoms Scale (DIEPSS) domains were examined. An external idiopathic Parkinson’s disease (IPD) dataset and machine learning were used as secondary analyses to evaluate diagnostic utility. **Results**: HHT-derived features showed vowel dependence, whereas nonlinear measures revealed broader abnormalities in DIP. Sample Entropy and the Correlation Dimension were most prominently increased in rounded back vowels /u/ and /o/. RQA determinism was significantly reduced across all vowels, with recurrence rate and laminarity reduced across multiple vowels, indicating impaired recurrence of phonation. Within the DIP cohort, dyskinesia showed the strongest association with acoustic abnormalities, whereas broader motor domains, including Gait, Bradykinesia, Rigidity, Tremor, Dyskinesia, and Overall Severity, characterized patient–control differences. Secondary analyses with Random Forest achieved a three-class classification of 76.0% (CI 70.3–81.3%). **Conclusions**: DIP is associated with measurable disruption of nonlinear vocal dynamics, characterized by vowel-sensitive HHT and entropy features and relatively consistent reductions in RQA determinism. These interpretable acoustic patterns may provide a non-invasive means of characterizing extrapyramidal motor dysfunction.

## 1. Introduction

Drug-Induced Parkinsonism (DIP) is one of the most common extrapyramidal symptoms (EPS) resulting from central dopamine *D*_2_ receptor blockade following antipsychotic therapy [1,2,3]. Clinically characterized by rigidity, bradykinesia, resting tremors, and gait disturbances, DIP often mimics idiopathic Parkinson’s disease (IPD), posing a major challenge for accurate differential diagnosis and side-effect monitoring. Traditional clinical assessment of DIP relies heavily on subjective physical examination scales, such as the Drug-Induced Extrapyramidal Symptom Scale (DIEPSS) [4,5,6] and the Glasgow Antipsychotic Side Effect Scale (GASS) [7]. While useful, these rating scales are time-consuming, prone to intra- and inter-rater variability, and unable to capture subtle, early stage motor dysfunctions before obvious movement impairment manifests [8].

In contrast, speech acoustic analysis could detect impairments in laryngeal motor control that may already be present at an early stage, with prior studies observing decreased variability in fundamental frequency during the prodromal period, detectable as early as five years prior to the diagnosis of Parkinson’s disease [9,10,11]. Interestingly, a similar classification study further supports this possibility, using speech feature extraction and machine learning algorithms to identify dysarthria-related patterns in longitudinal recordings of celebrities, they achieved a fair ROC-AUC of 0.75 five years prior to diagnosis [12]. In brief, patient speech could potentially provide a sensitive, non-invasive probe of extrapyramidal disorders. This may be attributed to the fine-grained coordination required for laryngeal motor control. In PD, for example, this control can be disrupted by abnormalities at multiple levels, including anatomical changes such as vocal-fold hyperadduction, muscular dysfunction characterized by rigidity and bradykinesia, and neurological dysfunction involving both dopaminergic and non-dopaminergic pathways [13], including laryngeal somatosensory deficits [14], which together may influence phonation, articulation, and prosody [15].

Over the past decade, advances in machine learning (ML) and deep learning have driven numerous studies toward the development of speech and voice-based diagnostic tools for Parkinson’s disease and other speech disorders [12,16,17,18,19,20,21,22,23,24]. Many of these approaches rely on conventional acoustic descriptors, including pitch perturbation (jitter), amplitude perturbation (shimmer), fundamental frequency (*F*_0_), and spectral representations such as Mel-Frequency Cepstral Coefficients (MFCCs). Although these features have demonstrated considerable value for classification, their physiological and mechanistic interpretability remains limited. In particular, MFCCs provide an efficient compact representation of the spectral envelope, but discriminative MFCC patterns do not readily translate into specific alterations in vocal-fold mechanics or the underlying nonlinear and non-stationary dynamics of phonation [16,22,24].

Voice production is inherently a complex dynamical process governed by interactions among aerodynamic forces, vocal-fold tissue oscillation, and neuromuscular control. When neurogenic impairment disrupts smooth vocal-fold mechanics, the laryngeal system shifts from stable periodic vibration toward nonlinear turbulence and chaotic dynamics, giving rise to complex recurrence structures and fractal scaling [25]. Capturing these underlying physical mechanisms requires advanced nonlinear signal processing methods. For instance, the Hilbert–Huang Transform (HHT), combining Empirical Mode Decomposition (EMD) and Hilbert Spectral Analysis, is particularly suitable for non-stationary signals, decomposing complex acoustic waveforms into intrinsic frequency components (IMFs) without imposing fixed basis functions [26]. Recent studies have applied HHT-derived features to Parkinson’s disease and voice pathology classification [27,28]. However, these approaches have largely emphasized time-dependent HHT feature sequences combined with deep-learning classifiers, thereby prioritizing predictive performance while retaining limited direct interpretability of the underlying acoustic dynamics.

An alternative approach is to derive compact, time-independent summary measures from HHT that characterize physically meaningful properties of the decomposed signal, such as frequency variability, spectral distribution, and relative energy organization. When integrated with nonlinear dynamic measures, including Correlation Dimension (*D*_2_), Largest Lyapunov Exponent (*λ*_1_), Sample Entropy, and Recurrence Quantification Analysis (RQA), such features may provide a more interpretable description of vocal-system instability, including attractor complexity, trajectory divergence, irregularity, and recurrent or laminar behavior [25,26]. This framework shifts the emphasis from identifying which high-dimensional features best classify disease toward understanding how the underlying vocal dynamics are altered.

Despite growing interest in HHT and nonlinear acoustic analysis [25,26,27,28], comprehensive integration of interpretable HHT-derived summary measures with quantified chaotic and phase-space dynamics remains scarce. This gap is particularly evident in drug-induced parkinsonism (DIP), for which acoustic markers of laryngeal instability remain substantially less studied than in idiopathic Parkinson’s disease (IPD). Given known clinical differences between DIP and IPD, including differences in resting tremors and motor asymmetry, it remains unclear whether these conditions exhibit distinct patterns of vocal nonlinearity, recurrence, and phase-space dynamics. Furthermore, acoustic changes across different degrees of extrapyramidal symptom severity, as quantified by the Drug-Induced Extrapyramidal Symptoms Scale (DIEPSS), remain poorly characterized. Addressing these gaps may therefore improve not only diagnostic discrimination but also the mechanistic interpretation of how different forms and severities of parkinsonism alter laryngeal motor dynamics.

To bridge this gap, this study proposes a physics-grounded, nonlinear acoustic framework to evaluate laryngeal stability in DIP. By combining HHT-derived spectral metrics with phase-space chaos parameters across five cardinal vowels (a, i, u, e, o), we aim to fulfill the following aims. First evaluate nonlinear dynamic degradation and energy redistribution of vocal-fold vibration across healthy controls (HC), DIP patients, and IPD patients; second, isolate key nonlinear physical parameters capable of capturing laryngeal micro-tremors, glottal instability, and phase-space trapped states; third, analyze overall marker significance on subcategories of the DIEPSS to uncover which symptom dimensions are most strongly associated with acoustic alterations; and lastly, develop a physics-grounded machine learning model for accurate and interpretable differential diagnosis between healthy, IPD, and DIP cohorts.

## 2. Materials and Methods

### 2.1. Dataset

To investigate Drug-Induced Parkinsonism (DIP), vocal recordings were collected from DIP patients with schizophrenia and healthy controls at Jianan Psychiatric Center, Taiwan. The DIP group comprised 82 patients with DSM-5 schizophrenia receiving antipsychotic treatment who fulfilled the study criteria for drug-induced parkinsonism, as determined through the DIEPSS, by board certified psychiatrists. The mean daily antipsychotic dose, expressed as a chlorpromazine-equivalent dose, was 522.17 ± 243.21 mg/day. Each participant produced sustained phonations of the five vowels (/a/, /i/, /u/, /e/, and /o/) at a sampling rate of *f_s_* = 11,025 Hz.

The patient group included 45 males and 37 females, whereas the healthy-control group included 14 males and 16 females. Sex distribution did not differ significantly between groups (χ^2^ = 0.25, *p* = 0.61). The mean age was 45.6 ± 10.2 years in the patient group and 30.8 ± 9.0 years in the healthy-control group, representing a significant between-group difference (F = 49.7, *p* < 0.01). The mean age at onset of schizophrenia in the patient group was 21.51 ± 8.02 years.

For secondary analysis with Idiopathic Parkinson’s Disease (IPD), an external dataset comprising recordings from patients with IPD (N = 560) and healthy controls (N = 574) was obtained from Kaggle [29] with a sampling rate of *f_s_* = 8000 Hz. This dataset was restricted to sustained /a/ phonations. Because the DIP and IPD cohorts originated from different datasets, control-referenced baseline normalization was performed prior to cross-disease comparison to reduce dataset-related variability.

### 2.2. Signal Preprocessing and Windowing

All voice recordings were uniformly resampled via librosa 0.11.0 module in python 3.13.14 to 11,025 Hz. To isolate steady-state vocal-fold dynamics from onset/offset acoustic transients, raw audio signals were preprocessed using a trimming procedure, trimming both silence and onset/offset vocals (200 ms); then, we normalized the amplitude. The data were separated by three sequential non-overlapping temporal windows with a length of 2 s.

### 2.3. Hilbert–Huang Transform (HHT) and Ensemble EMD

**Figure 1:**
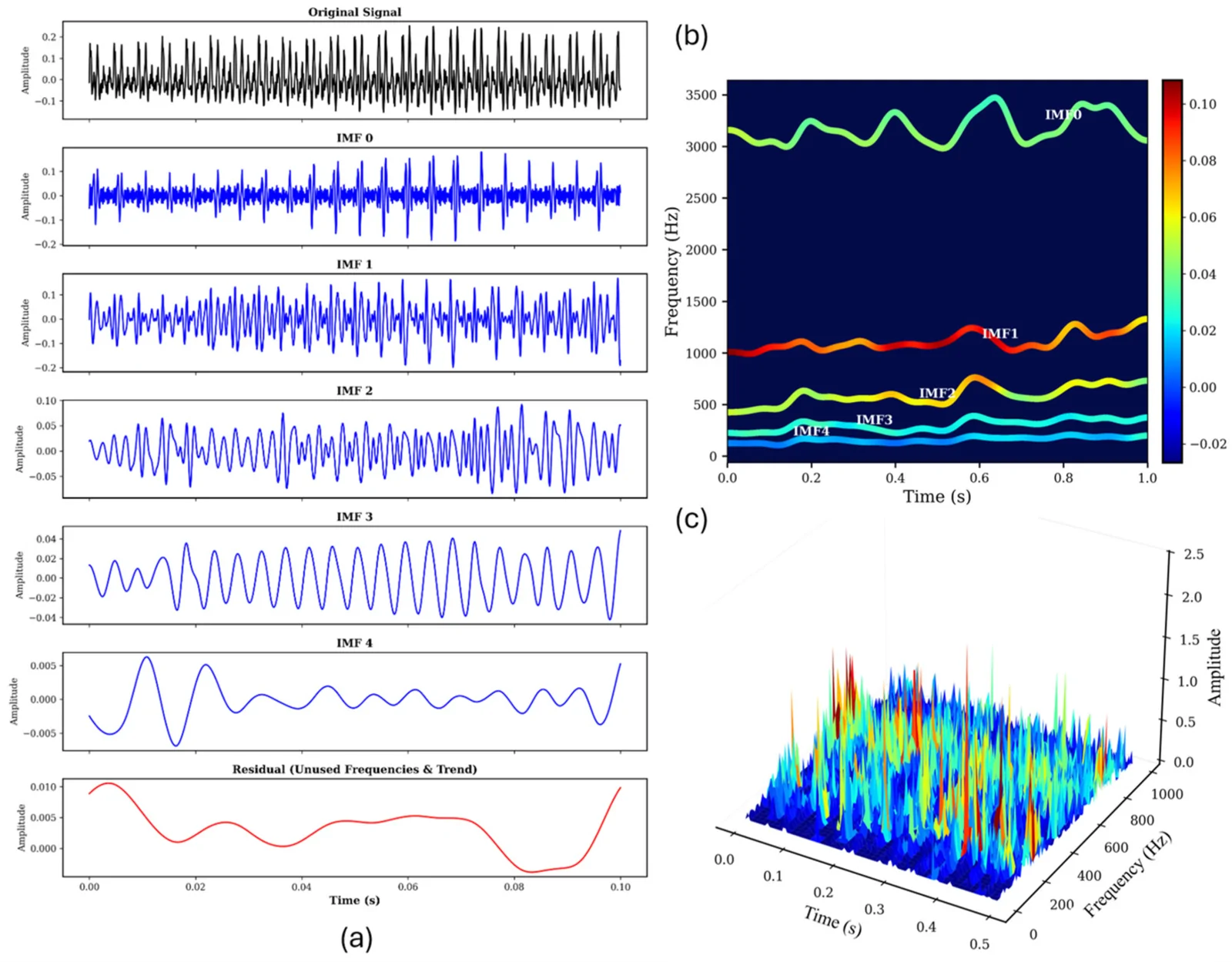
(**a**) EEMD Mode Decomposition of Patient Vocals; (**b**) Gaussian-Filtered Instantaneous Frequency of IMFs; (**c**) Dynamic Frequency–Energy Plot, colors denote energy intensity.

The non-stationary and nonlinear characteristics of vocal-fold vibration were decomposed using Ensemble Empirical Mode Decomposition (EEMD). Five intrinsic mode functions (IMFs; IMF0–IMF4) were retained to provide a consistent decomposition across participants while capturing the principal frequency components of sustained phonation. These IMFs spanned from lower-frequency components encompassing the fundamental-frequency range (IMF4: approximately 60–280 Hz) to higher-frequency harmonic, formant, and perturbation components (IMF0: >3000 Hz). The empirical frequency distributions of each IMF across all five vowels are provided in Appendix C.1 (Table A7), and representative EEMD decompositions are illustrated in Figure 1.

#### 2.3.1. EEMD

For each discrete windowed signal *x*[*n*], EEMD generates *E* = 100 independent realizations by adding zero-mean Gaussian white noise $v_{e}\left[n\right]∼N\left(0,\sigma_{v}^{2}\right)$ to prevent mode mixing:

(1) $$\begin{array}{c} x_{e}\left[n\right]=x\left[n\right]+v_{e}\left[n\right],e=1,2,\ldots ,E, \end{array}$$

where *σ_v_* is parameterized with an amplitude factor of *ϵ* = 0.05 (5%)

(2) $$\begin{array}{c} \sigma_{v}=\epsilon \cdot \left(\max_{n}x\left[n\right]-\min_{n}x\left[n\right]\right),\; \end{array}$$

Each trial *x_e_*[*n*] is decomposed into *M* = 5 Intrinsic Mode Functions (IMFs), *c_m_*_,*e*_[*n*], and a monotonic residual *r_e_*[*n*] using the sifting process:

(3) $$\begin{array}{c} x_{e}\left[n\right]=\sum_{m=0}^{M-1}c_{m,e}\left[n\right]+r_{e}\left[n\right],\; \end{array}$$

The final ensemble-averaged IMFs ${\bar{c}}_{m}\left[n\right]$ are computed as follows:

(4) $$\begin{array}{c} {\bar{c}}_{m}\left[n\right]=\frac{1}{E}\sum_{e=1}^{E}c_{m,e}\left[n\right],m=0,1,2,\ldots ,M-1,\; \end{array}$$

#### 2.3.2. Hilbert Spectral Analysis

The analytic signal *z_m_*[*n*] for each IMF ${\bar{c}}_{m}\left[n\right]$ is formulated via the Hilbert Transform H{⋅}:

(5) $$\begin{array}{c} z_{m}\left[n\right]={\bar{c}}_{m}\left[n\right]+jH\left\{{\bar{c}}_{m}\left[n\right]\right\}=a_{m}\left[n\right]e^{j\phi_{m}\left[n\right]},\; \end{array}$$

where the instantaneous amplitude *a_m_*[*n*] and instantaneous phase *ϕ_m_*[*n*] are defined by:

(6) $$\begin{array}{c} a_{m}\left[n\right]=\sqrt{{\bar{c}}_{m}^{2}\left[n\right]+H^{2}\left\{{\bar{c}}_{m}\left[n\right]\right\}},\; \end{array}$$

(7) $$\begin{array}{c} \phi_{m}\left[n\right]=\mathrm{unwrap}\left(\arctan\left(\frac{H\left\{{\bar{c}}_{m}\left[n\right]\right\}}{{\bar{c}}_{m}\left[n\right]}\right)\right), \end{array}$$

The discrete instantaneous frequency *ω_m_*[*n*] (in Hz) is derived from the phase derivative:

(8) $$\begin{array}{c} \omega_{m}\left[n\right]=\frac{f_{s}}{2\pi }\left(\phi_{m}\left[n+1\right]-\phi_{m}\left[n\right]\right),\; \end{array}$$

#### 2.3.3. Derived HHT Feature Formulations

For each IMF *m* ∈ {0,1,2,3,4}, three specific metrics are extracted, together with a global high-to-low energy ratio:

- Frequency Variance ($\sigma_{\omega ,m}^{2}$):
  (9) $$\begin{array}{c} \sigma_{\omega ,m}^{2}=\frac{1}{N_{w}-1}\sum_{n=0}^{N_{w}-1}{\left(\omega_{m}\left[n\right]-{\bar{\omega }}_{m}\right)}^{2}, \end{array}$$
  where
  (10) $$\begin{array}{c} {\bar{\omega }}_{m}=\frac{1}{N_{w}-1}\sum_{n=1}^{N_{w}-1}\omega_{m}\left[n\right]\;,\; \end{array}$$
- Frequency Spectral Entropy (*H_ω_*_,*m*_): Constructing a 50-bin normalized probability density function *P_m_*(*b*) of *ω_m_*[*n*] over non-zero bins *B*:
  (11) $$\begin{array}{c} H_{\omega ,m}=-\sum_{b\in B}P_{m}\left(b\right){\log_{2}P}_{m}\left(b\right),\; \end{array}$$
- IMF Energy Ratio (*E*_ratio,*m*_): Defining IMF energy $E_{m}=\sum_{n=0}^{N_{w}-1}a_{m}^{2}\left[n\right]$:
  (12) $$\begin{array}{c} E_{\mathrm{ratio},m}=\frac{E_{m}}{\sum_{k=0}^{M-1}E_{k}+\epsilon }, \end{array}$$
- High-to-Low Energy Ratio (*R*_HL_):
  (13) $$\begin{array}{c} R_{\mathrm{HL}}=\frac{E_{0}+E_{1}}{\sum_{m=2}^{M-1}E_{m}+\epsilon }, \end{array}$$

### 2.4. Nonlinear Dynamics and Chaos Quantification

**Figure 2:**
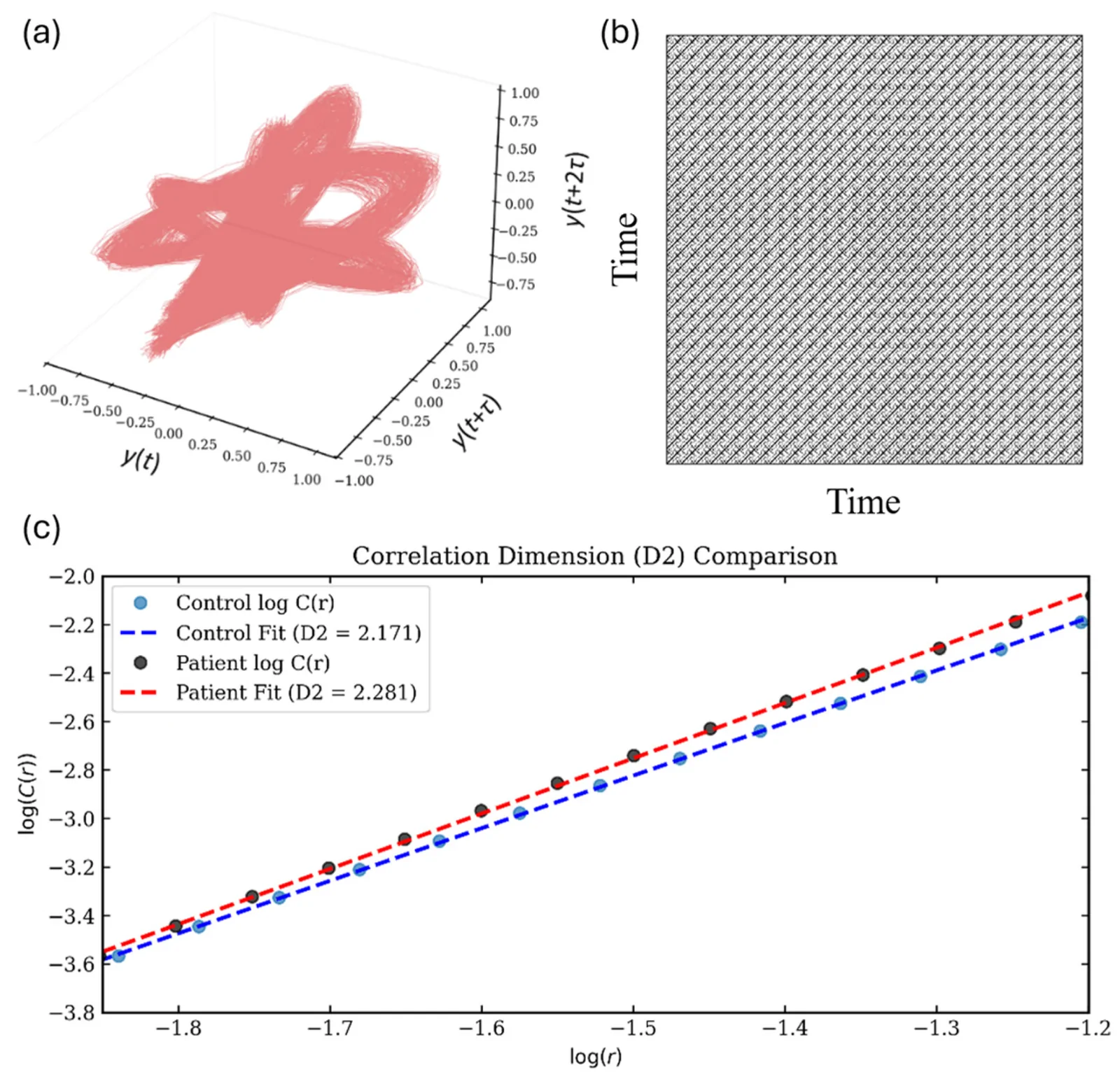
(**a**) Representative phase-space reconstruction of a DIP vocal signal showing trajectory divergence, *m* = 3, *τ* = 15 (1.36 ms) for visualization; (**b**) recurrence plot of DIP patient vocals; (**c**) representative comparison of correlation-dimension estimation between a DIP participant and a healthy control.

To characterize nonlinear laryngeal instability, a centered sub-segment *s*[*n*] of length *N_s_* = 10,000 samples (*T_s_* ≈ 0.9 s) is mapped into an *m*-dimensional phase-space attractor using Takens’ Delay Embedding Theorem [30] and computed via the python *NeuroKit2 0.2.13* module. The visualizations are shown in Figure 2.

#### 2.4.1. Phase-Space Reconstruction

Given embedding dimension *m* and time delay *τ*, the trajectory matrix $Y\in R^{N_{p}\times m}$ is constructed, where *N_p_* = *N_s_* − (*m* − 1)*τ* denotes the total number of reconstructed state vectors:

(14) $$\begin{array}{c} y_{i}={\left[s,\left[i\right],s,\left[i+\tau \right]\ldots s\left[i+\left(m-1\right)\tau \right]\right]}^{T}\in R^{m},\;i=1,2,\ldots ,N_{p}\; \end{array}$$

Signal-specific embedding parameters were estimated independently for each vocal segment: the optimal delay *τ* was identified using the first local minimum of the Average Mutual Information (AMI) function [31], and the minimum embedding dimension *m* was determined using the False Nearest Neighbors (FNN) algorithm [32].

#### 2.4.2. Correlation Dimension (*D*_2_)

The Correlation Dimension (*D*_2_) characterizes the geometric complexity and fractal dimensionality of the reconstructed attractor. Using the Grassberger-Procaccia algorithm [33], the spatial correlation integral *C*(*r*) measures the relative frequency of vector pairs closer than a spatial threshold *r* under the Euclidean distance metric

(15) $$\begin{array}{c} d_{2}\left(y_{i},y_{j}\right)=∥y_{i}-y_{j}{∥}_{2}:C\left(r\right)=\frac{2}{N_{p}\left(N_{p}-1\right)}\sum_{i=1}^{N_{p}}\sum_{j=i+1}^{N_{p}}\Theta \left(r-∥y_{i}-y_{j}{∥}_{2}\right), \end{array}$$

where Θ(⋅) represents the Heaviside step function, evaluated on the upper-triangular distance matrix (*j* > *i*) to exclude self-correlations. In practice, *C*(*r*) is evaluated across 64 geometrically spaced radius thresholds spanning 2.5% to 50% of the attractor’s pairwise distance range. The Correlation Dimension *D*_2_ is estimated as the slope of the linear least-squares regression line in the log-log scaling region:

(16) $$\begin{array}{c} D_{2}=\lim_{r\to 0}\frac{\partial \ln C\left(r\right)}{\partial \ln r}\approx \frac{\Delta \ln C\left(r\right)}{\Delta \ln r} \end{array}$$

#### 2.4.3. Largest Lyapunov Exponent (*λ*_1_)

To quantify the rate of dynamic divergence and sensitivity to initial conditions, the Largest Lyapunov Exponent (*λ*_1_) was estimated via Rosenstein’s algorithm [34]. For each reference vector *y_j_*, its nearest neighbor $y_{\hat{j}}$ is identified subject to a temporal separation constraint (Theiler window, *W*):

(17) $$\begin{array}{c} |j-\hat{j}|\;>\;W\;, \end{array}$$

where *W* is dynamically parameterized as the mean period of the phonation (*W* = ⌈*f_s_*/*f*_mean_⌉) to prevent spurious temporal correlations between points on the same trajectory. The divergence distance $d_{j}\left(i\right)\;=\;∥y_{j+i}-y_{\hat{j}+i}{∥}_{2}$ between neighboring trajectories is then tracked over discrete time steps *i*.

Assuming exponential divergence, *λ*_1_ is computed as the slope of the linear least-squares fit across the average logarithmic divergence curve ⟨ln*d_j_*(*i*)⟩ over the initial linear divergence horizon (*i* = 1,2,…,20 samples):

(18) $$\begin{array}{c} \left\langle \ln d_{j}\left(i\right)\right\rangle \approx \lambda_{1}\cdot \left(i\cdot \Delta t\right)+\left\langle \ln d_{j}\left(0\right)\right\rangle \end{array}$$

#### 2.4.4. Sample Entropy (SampEn)

Defining *B^m^*(*r*) as the probability that two sequences of length *m* match within tolerance *r* = 0.2 ⋅ std(*s*), and *A^m^*^+1^(*r*) as the match probability for length *m* + 1:

(19) $$\begin{array}{c} B^{m}\left(r\right)=\frac{1}{N_{s}-m}\sum_{i=1}^{N_{s}-m}\left(\frac{1}{N_{s}-m-1}\sum_{j=1,j\neq i}^{N_{s}-m}\Theta \left(r-\|x_{m}\left(i\right)-x_{m}\left(j\right)\|\infty \right)\right),\; \end{array}$$

(20) $$\begin{array}{c} \mathrm{SampEn}\left(m,r,N_{s}\right)=-\ln\left(\frac{A^{m+1}\left(r\right)}{B^{m}\left(r\right)}\right), \end{array}$$

### 2.5. Recurrence Quantification Analysis (RQA)

Using the normalized phase-space trajectory Y, the unthresholded distance matrix *D_i_*_,*j*_ = ‖*y_i_* − *y_j_*‖_∞_ was converted into a binary recurrence matrix using *ε* = 0.1, corresponding to 5% of the normalized maximum attractor distance:

(21) $$\begin{array}{c} R_{i,j}=\Theta \left(\varepsilon -\|y_{i}-y_{j}\|\infty \right),i,j=1,2,\ldots ,N_{p},\; \end{array}$$

The structural topology of R is quantified through three RQA metrics.

- Recurrence Rate (RR): The proportion of recurrent points in phase space:
  (22) $$\begin{array}{c} \mathrm{RR}=\frac{1}{N_{p}^{2}}\sum_{i=1}^{N_{p}}\sum_{j=1}^{N_{p}}R_{i,j}, \end{array}$$
- Determinism (DET): The fraction of recurrence points that form diagonal lines of minimum length *l*_min_ = 2, reflecting deterministic trajectory predictability:
  (23) $$\begin{array}{c} \mathrm{DET}=\frac{\sum_{l=l_{\min}}^{N_{p}}l\;P\left(l\right)}{\sum_{i=1}^{N_{p}}\sum_{j=1}^{N_{p}}R_{i,j}}, \end{array}$$
  where *P*(*l*) is the frequency distribution of diagonal lengths of length *l*.
- Laminarity (LAM): The proportion of recurrence points forming vertical structures of minimum length *v*_min_ = 2, quantifying phase-space trapped states:
  (24) $$\begin{array}{c} \mathrm{LAM}=\frac{\sum_{v=v_{\min}}^{N_{p}}v\;P\left(v\right)}{\sum_{v=1}^{N_{p}}v\;P\left(v\right)}, \end{array}$$
  where *P*(*v*) is the frequency distribution of vertical lengths of length *v*.

Note that prior to recurrence analysis, distances within each reconstructed phase-space attractor were normalized, yielding a common normalized maximum distance of 2. Accordingly, the recurrence threshold ε = 0.1 corresponds to 5% of the normalized maximum attractor distance and therefore represents a dimensionless scaled threshold rather than an absolute threshold in the original phase-space coordinates, thus enabling comparisons across participants, vowels, and reconstructed attractors with different embedding dimensions. Robustness to threshold selection was further evaluated across ε = 0.05–0.15 (Appendix C.2) [35].

### 2.6. Statistical Analysis

All statistical analyses were conducted using Python 3.13.14 with the SciPy 1.15.3 and Pandas 2.3.3 libraries. To ensure the assumption of independent observations, all window-bootstrapped acoustic features were first aggregated by calculating the mean for each unique participant.

Four primary statistical comparisons were performed:

- DIP vs. Healthy Controls: To identify acoustic features significantly altered by Drug-Induced Parkinsonism. The full results are provided in File S4.
- DIP-DIEPSS Correlations: To assess how acoustic degradation maps onto specific clinical phenotypes, Spearman rank correlations were computed across all acoustic features (N = 110 metrics) for each individual DIEPSS subcategory (items 1–9). To evaluate panel-wide signal enrichment across multiple acoustic features, empirical *p*-value distributions were visualized via Quantile–Quantile (Q-Q) plots against the theoretical uniform null distribution. Distribution inflation factors based on both the median (*λ_median_*) and mean (*λ_mean_*) of the test statistics were calculated to identify symptom domains exhibiting systematic, widespread association with DIP laryngeal dysfunction. The full results are provided in Files S2 and S3.
- IPD vs. Healthy Controls: To identify acoustic features significantly altered by Idiopathic Parkinson’s Disease. The full results are provided in File S6.
- DIP vs. IPD: To enable direct DIP–IPD comparison across distinct datasets, features were standardized against their respective healthy controls via Z-score normalization. This converted the raw acoustic values into standardized Z-scores, thereby reducing, but not eliminating, potential dataset-related scale differences. Group differences across the 22 acoustic features were tested using 10,000 permutations and corrected using the Benjamini–Hochberg FDR procedure. The full results are provided in File S5.

Because nonlinear acoustic features frequently violate assumptions of normality, the non-parametric Mann–Whitney U test (two-sided) was utilized for all comparisons to determine statistical significance. To quantify the clinical magnitude of these differences independent of sample size, Cohen’s d was calculated using the pooled standard deviation. Features were subsequently ranked descriptively according to statistical significance and effect size to summarize the relative magnitude of between-group differences. To assess the robustness of the findings, supplementary sensitivity analyses were performed using age- and sex-adjusted regression models, with Benjamini–Hochberg false discovery rate (FDR) correction across the 20 prespecified vowel–feature comparisons. Detailed results are provided in Appendix A.1 and Appendix A.2, with raw data provided in File S1.

### 2.7. Multi-Vowel Combinatorial Fusion and Cross-Validation

To construct subject-level feature representations without introducing artificial temporal dependencies, a combinatorial sampling strategy is applied:

1. Vowel Feature Assembly: For subject *S_u_* and vowel *v* ∈ {a, i, u, e, o}, window features are stored in set $F_{u,v}$.
2. Bootstrapped Feature Fusion: Exactly 10 synthetic subject-level feature vectors $x_{u}^{\left(g\right)}$ (*g* = 1,…,10) are formed by randomly sampling one window vector per vowel:
  (25) $$\begin{array}{c} x_{u}^{\left(g\right)}=\left[f_{u,a}^{\left(g\right)}\;\|\;f_{u,i}^{\left(g\right)}\;\|\;f_{u,u}^{\left(g\right)}\;\|\;f_{u,e}^{\left(g\right)}\;\|\;f_{u,o}^{\left(g\right)}\right], \end{array}$$
  where
  (26) $$\begin{array}{c} f_{u,v}^{\left(g\right)}∼\mathrm{Uniform}\left(F_{u,v}\right), \end{array}$$
3. Validation Integrity: To strictly prevent data leakage resulting from subject-level bootstrapping, all classification experiments utilize Group *K*-Fold Cross-Validation (*K* = 4), where all 10 bootstrapped rows belonging to subject *S_u_* are strictly restricted to either the training set or the validation set within any given fold. Predictions are generated exclusively on the held-out validation subjects per fold, producing an un-biased Out-of-Fold (OOF) prediction matrix across the entire dataset for downstream evaluation.

### 2.8. Machine Learning and Model Evaluation

To evaluate the diagnostic utility of the extracted nonlinear acoustic features, machine learning models were developed using Random Forest (RF) and eXtreme Gradient Boosting (XGBoost) algorithms. These tree-based algorithms were selected for their inherent robustness against nonlinear data distributions and their ability to model complex feature interactions without requiring extensive data scaling.

Three distinct classification tasks were designed:

- DIP vs. Healthy: A binary classifier trained to distinguish patients with Drug-Induced Parkinsonism from healthy controls.
- IPD vs. Healthy: A binary classifier trained to distinguish patients with Idiopathic Parkinson’s Disease from healthy controls.
- Multiclass Ensemble Classifier (DIP vs. IPD vs. Healthy): A multiclass framework designed to categorize an acoustic profile into one of the three clinical states.

A significant methodological challenge in merging these cohorts was class imbalance, owing to the large size of the IPD and Healthy datasets relative to the DIP cohort. To fully utilize larger datasets without inducing predictive bias toward the majority classes, a multiclass ensemble approach comprising 7 independent models was developed. For each of the 7 models, the IPD and Healthy cohorts were randomly under-sampled to exactly match the size of the DIP cohort, guaranteeing a perfectly balanced 1:1:1 training ratio. The final multiclass prediction was derived by aggregating the outputs of these 7 models via majority voting.

### 2.9. Model Validation

Model performance was comprehensively evaluated on the concatenated Out-of-Fold (OOF) predictions using standard classification metrics, including Accuracy, Precision, Recall (Sensitivity), *F*_1_-Score, and the Area Under the Receiver Operating Characteristic Curve (ROC-AUC). Nested hyperparameter optimization was conducted for RF and XGBoost, with validation ROC-AUC and accuracy serving as the objective metrics for binary and multiclass tasks, respectively. The exact parameters were detailed in Appendix B. Subject-level probability scores were aggregated across the 10 bootstrapped samples per subject to yield final subject-level ROC-AUC and diagnostic precision.

To quantify uncertainty in classification performance, 95% confidence intervals were estimated using 1000 bootstrap resamples of the out-of-fold predictions. In addition, non-parametric permutation testing with 1000 random label permutations was performed separately for the DIP–HC and IPD–HC classification tasks to determine whether the observed model performance exceeded that expected under random label assignment. These analyses assess above-chance classification performance but do not exclude residual demographic or dataset-specific confounding.

## 3. Results

### 3.1. Hilbert–Huang Transform Markers

**Figure 3:**
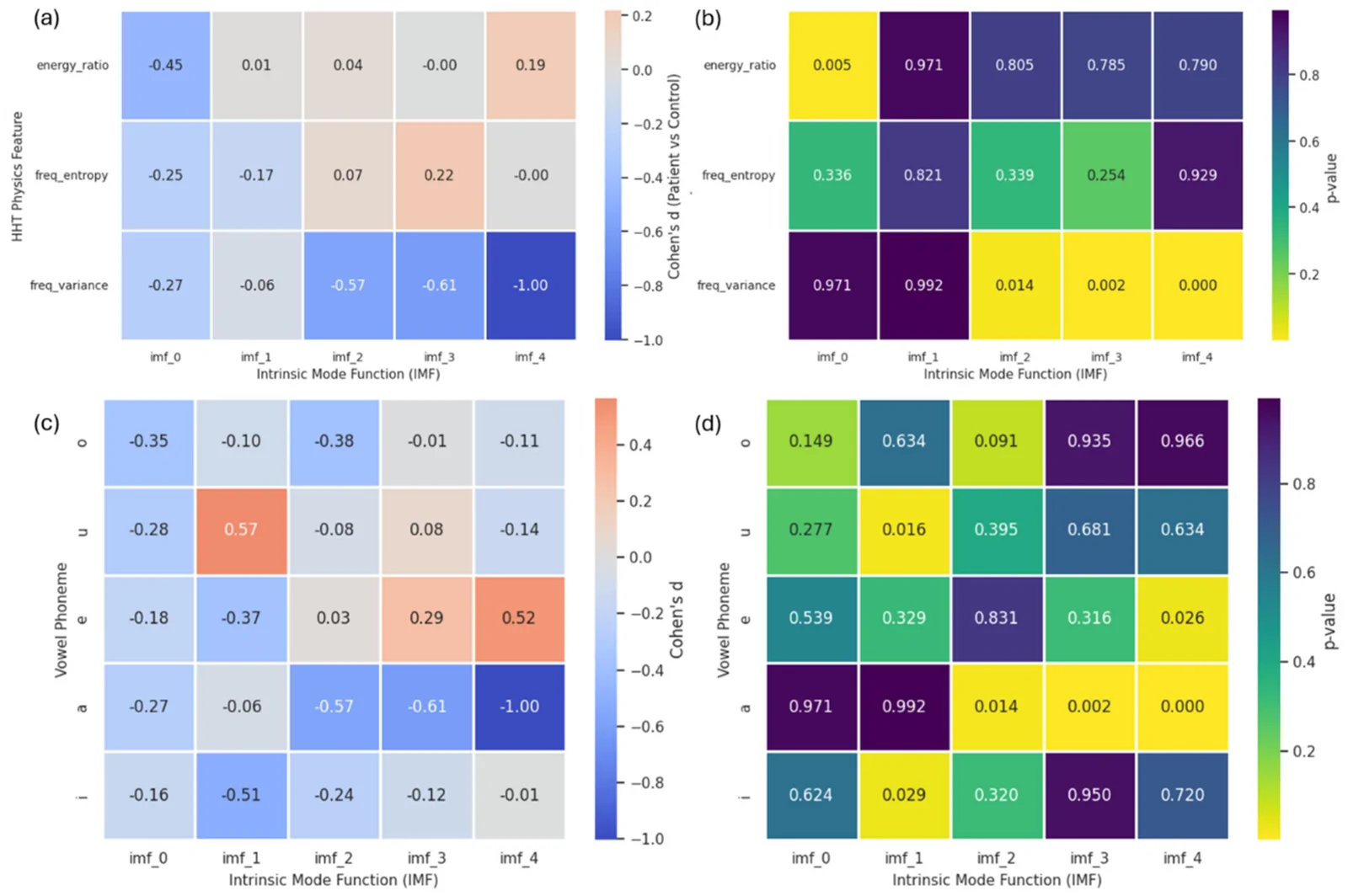
Hilbert–Huang Transform (HHT) physics-based feature analysis between DIP patients and healthy controls: (**a**) Cohen’s d effect sizes for vowel /a/ features across IMFs; (**b**) corresponding *p*-values for vowel /a/ features; (**c**) Cohen’s d effect sizes for frequency variance across all five cardinal vowels and IMFs; (**d**) corresponding *p*-values for frequency variance across vowels and IMFs.

As illustrated in Figure 3a,b, a statistically significant difference emerges in the lower-frequency Intrinsic Mode Function (IMF) regimes for sustained vowel /a/. Specifically, IMF 4, IMF 3, and IMF 2 exhibit higher values in healthy controls (*p* = 4.56 × 10^−5^, *p* = 0.002, and *p* = 0.014, respectively). This indicates that the variance of low-frequency oscillations is decreased in DIP, likely reflecting vocal-fold motor restriction and laryngeal rigidity in patients.

However, a broader investigation across all vowel phonemes in Figure 3c,d reveals no consistent or universal trend in the HHT metrics between cohorts. Because empirical mode decomposition characteristics depend inherently on the acoustic properties of the produced sound, vowel-specific articulatory dynamics exert distinct effects on the extracted physical metrics. This highlights the role of HHT sound characteristics as a vowel-dependent augmentation marker and underscores the diagnostic superiority of universal nonlinear markers including RQA and Sample Entropy, which we will discuss in the next section.

### 3.2. Nonlinear System Markers

#### 3.2.1. DIP vs. HC

**Figure 4:**
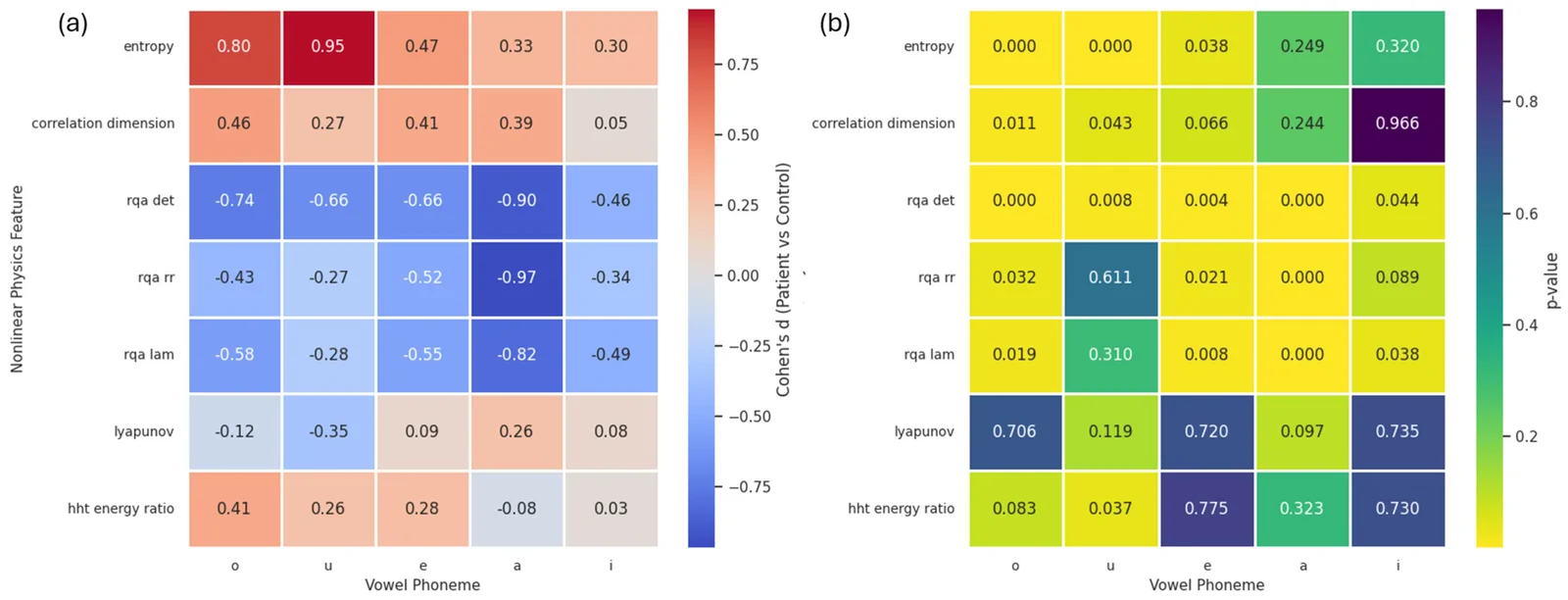
(**a**) Heatmap of Vowel Nonlinear Physics Feature Cohen’s D Values between DIP Patients and Healthy Controls; (**b**) Heatmap of Vowel Nonlinear Physics Feature *p*-Values between DIP Patients and Healthy Controls.

In Figure 4a,b, a comprehensive evaluation of nonlinear dynamics and phase-space metrics reveals significant laryngeal motor degradation in patients with Drug-Induced Parkinsonism (DIP) compared to healthy controls across multiple cardinal vowels. Unlike the vowel-dependent variations observed in frequency-domain HHT features, key nonlinear measures, specifically Sample Entropy, Correlation Dimension (*D*_2_), and Recurrence Quantification Analysis (RQA) metrics, demonstrate remarkable consistency and diagnostic utility across phoneme contexts.

A striking increase in vocal dynamic complexity and irregularity is observed in DIP patients. Sample Entropy exhibits substantial positive effect sizes across all phonemes, reaching statistical significance in vowels /o/ (*d* = 0.80, *p* < 0.001), /u/ (*d* = 0.95, *p* < 0.001), and /e/ (*d* = 0.47, *p* = 0.038), reflecting an increase in aperiodic, turbulent vocal-fold vibration secondary to incomplete glottal closure and impaired muscle tone control.

Concurrently, phase-space attractor complexity, quantified via the Correlation Dimension (*D*_2_), is consistently elevated in the DIP cohort. As illustrated in Figure 2c and Figure 4a,b, DIP patients exhibit a higher spatial embedding dimension across vowels /o/ (*d* = 0.46, *p* = 0.011) and /u/ (*d* = 0.27, *p* = 0.043). This increase in *D*_2_ indicates a higher number of active degrees of freedom governing vocal-fold oscillation, confirming that neurogenic disruption in DIP shifts smooth, low-dimensional glottal attractors toward higher-dimensional chaotic states.

From a vocal tract aerodynamic perspective, the heightened nonlinearity observed in high-to-mid back rounded vowels, specifically /o/ and /u/, can be attributed to the acoustic coupling between the laryngeal source and the vocal tract configuration. As established in classical acoustic theory, back rounded vowels such as /o/ and /u/ require lip rounding and protraction, which constricts the anterior vocal tract exit and lengthens the overall tract cavity. This anterior constriction elevates acoustic impedance and supra-glottal backpressure, creating a semi-occluded vocal tract (SOVT) environment [36,37,38].

When the laryngeal musculature functions normally, this inertive reactance supports stable glottal self-oscillation. As detailed by Franca (2012) [37], high-back rounded vowels (/u/) and mid-back rounded vowels (/o/) demonstrate statistically significant reductions in shimmer and voice turbulence in healthy controls compared to front unrounded vowels, establishing back vowels as highly stable acoustic tasks in normative speech. However, under the neurogenic dysregulation and muscular rigidity characteristic of DIP, the added acoustic loading severely amplifies underlying glottal irregularities. Consequently, minor asymmetrical oscillations or incomplete glottal closures are translated into pronounced acoustic turbulence and chaotic path divergence, as summarized in Table 1.

Although entropy and correlation dimension metrics offer more intuitive physical insights into patient–control differences than HHT features, their statistical significance remains vowel-dependent. In contrast, Recurrence Quantification Analysis (RQA) metrics demonstrate the most robust diagnostic separation between DIP patients and healthy controls across phonemic contexts, with recurrence structures universally lower in patients.

Among the evaluated RQA metrics, a pronounced and statistically significant collapse in determinism (RQA DET) is observed across all five cardinal vowels: /a/ (d = −0.90, *p* < 0.001), /o/ (d = −0.74, *p* < 0.001), /e/ (d = −0.66, *p* = 0.004), /u/ (d = −0.66, *p* = 0.008), and /i/ (d = −0.46, *p* = 0.044). This sharp drop in diagonal line structures within reconstructed phase space signifies a fundamental loss of predictable, rule-governed vocal-fold periodicity, primarily driven by laryngeal micro-tremors and glottal instability.

Parallel to this deterministic degradation, vertical line recurrence structures (RQA LAM), which quantify phase-space intermittency and trapped states, are significantly reduced in patients with Drug-Induced Parkinsonism across /a/ (d = −0.82, *p* < 0.001), /o/ (d = −0.58, *p* = 0.019), /e/ (d = −0.55, *p* = 0.008), and /i/ (d = −0.49, *p* = 0.038). Furthermore, overall trajectory recurrence rate (RQA RR) is markedly diminished, reaching robust statistical significance in vowels /a/ (d = −0.97, *p* < 0.001), /e/ (d = −0.52, *p* = 0.021), and /o/ (d = −0.43, *p* = 0.032), collectively demonstrating a systemic breakdown of phase-space recurrence dynamics in pathological voice production.

#### 3.2.2. DIP vs. IPD

As shown in Table 2, compared with IPD, DIP exhibited a significantly lower recurrence rate (RR; *d* = −0.61, FDR-adjusted permutation *q* = 4.40 × 10^−4^), determinism (DET; *d* = −0.73, *q* = 4.40 × 10^−4^), and laminarity (LAM; *d* = −0.58, *q* = 4.40 × 10^−4^). Conversely, Sample Entropy (*d* = 0.52, *q* = 4.40 × 10^−4^) and Correlation Dimension (*d* = 0.31, *q* = 2.35 × 10^−2^) were significantly higher in DIP. The Lyapunov Exponent showed a small positive effect (*d* = 0.27; permutation *p* = 2.71 × 10^−2^), but this difference did not remain significant after FDR correction (*q* = 5.42 × 10^−2^). Overall, these findings indicate greater disruption of recurrent and regular vocal dynamics in the DIP cohort relative to the IPD cohort, together with increased nonlinear complexity.

Although DIP and IPD arise in distinct clinical contexts—pharmacological dopamine-receptor blockade and progressive nigrostriatal neurodegeneration, respectively—the present cross-dataset design does not permit these acoustic differences to be attributed directly to disease-specific neurobiological mechanisms. Importantly, the persistence of group differences in key nonlinear features after direct DIP–IPD label-permutation testing supports that the observed separation is unlikely to reflect random label assignment alone. Nevertheless, because disease status is confounded with dataset origin, residual effects of recording conditions, cohort characteristics, and differences in symptom severity cannot be excluded.

### 3.3. DIEPSS Comparison

**Figure 5:**
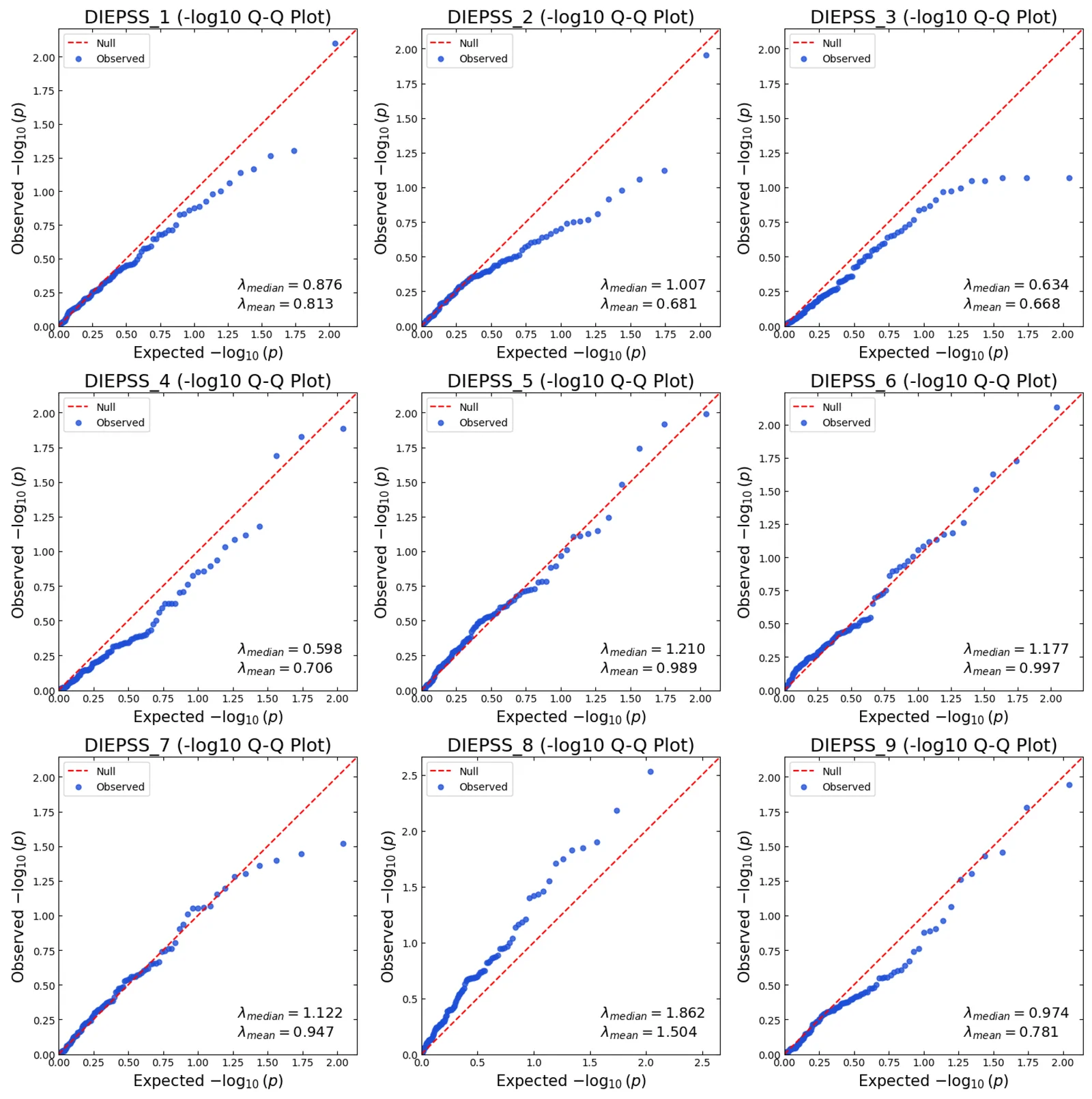
Q−Q Plot of acoustic metrics’ Spearman *p*-values across DIEPSS 1−9 within DIP.

The clinical severity of patients with drug-induced parkinsonism (DIP) was assessed using the Drug-Induced Extrapyramidal Symptoms Scale (DIEPSS), which comprises nine individual items: Gait, Bradykinesia, Sialorrhea, Rigidity, Tremor, Akathisia, Dyskinesia, Dystonia, and Overall Severity [4,5,6]. Each item is scored from 0 (normal) to 4 (severe) through objective observation and clinical interviews. To evaluate the association between the extracted acoustic metrics and clinical severity, Spearman rank correlations were computed across the DIP patient database and visualized using −log_10_ Quantile–Quantile (Q-Q) plots, with inflation factors calculated for both mean (*λ*_mean_) and median (*λ*_median_) to account for feature panel scale (*N* = 110 metrics; Figure 5).

As illustrated in Figure 5, for most DIEPSS items (Gait, Bradykinesia, Sialorrhea, Rigidity, Tremor, Akathisia, Dystonia and Overall Severity), the observed *p*-value distributions aligned closely with or fell below the null expectation line (*λ*_mean_ ≤ 1.0), reflecting an absence of widespread correlation with the acoustic metrics within the patient group. In contrast, Dyskinesia (DIEPSS-8) demonstrated a marked upward departure from the null line on the Q-Q plot, accompanied by substantial distribution-wide inflation (*λ*_median_ = 1.862, *λ*_mean_ = 1.504). Global significance testing confirmed that this systematic deviation was statistically significant (Fisher’s combined test, *p* = 0.0002; Kolmogorov–Smirnov test, *p* = 0.0014). This selective enrichment suggests that glottal dynamic instability among DIP patients is predominantly driven by dyskinetic manifestations.

**Figure 6:**
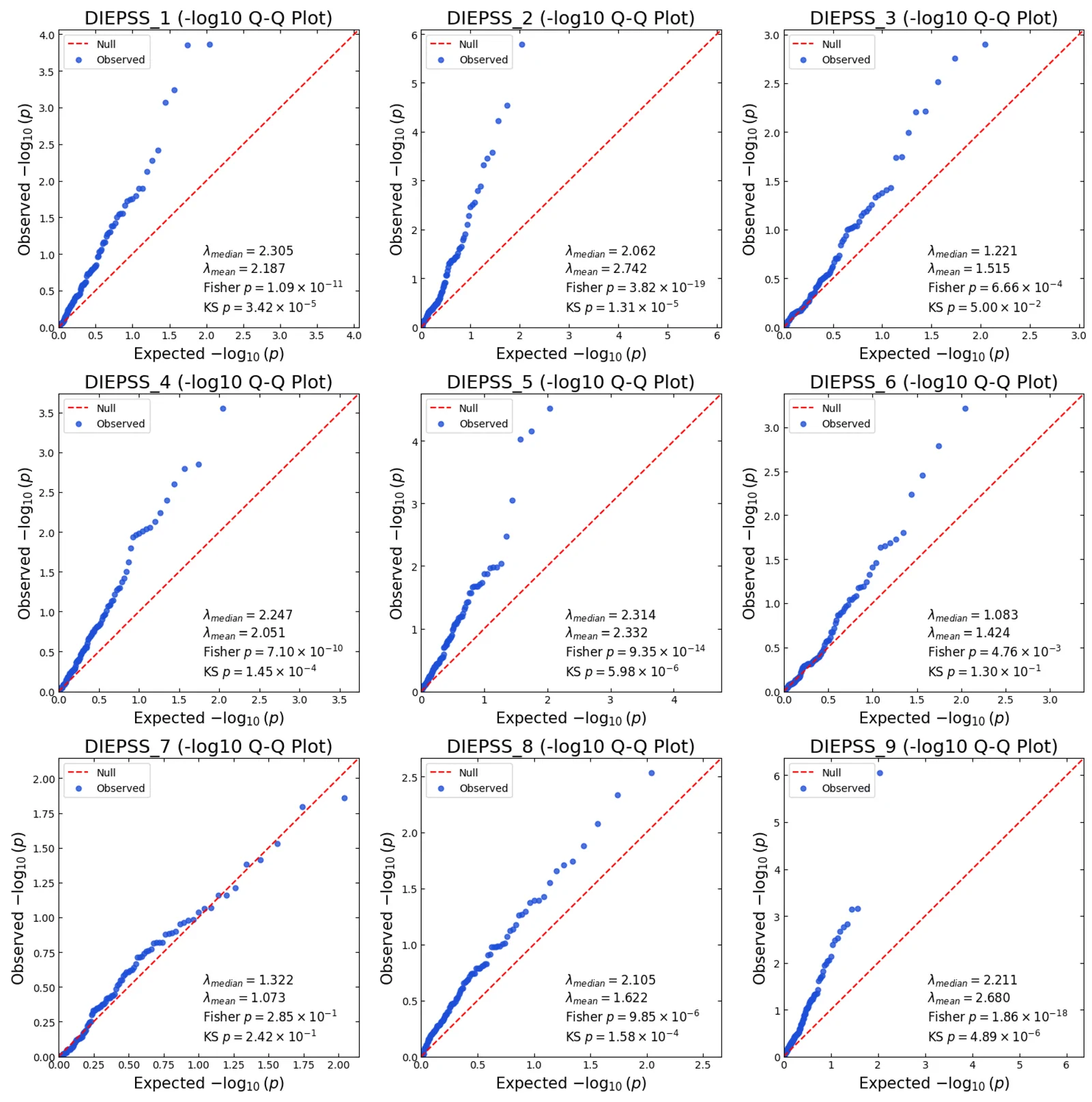
Q−Q Plot of acoustic metrics’ Spearman *p*−values across DIEPSS 1−9 within all participants (DIP and HC), with HC marked as zero (normal) in every sub-score.

In contrast, expanding the analysis across all participants, including healthy controls (HC) and patients with drug-induced parkinsonism (DIP), yielded markedly different results. As illustrated in Figure 6, sub-scores for DIEPSS 1, 2, 4, 5, 8, and 9 reached robust statistical significance across both Fisher’s combined probability test and the Kolmogorov–Smirnov (KS) test, with each demonstrating inflation factors (*λ*) exceeding 2. Specifically, marked deviations from the null distribution were observed in Gait (Fisher’s *p* = 1.09 × 10^−11^, KS *p* = 3.42 × 10^−5^), Bradykinesia (Fisher’s *p* = 3.83 × 10^−19^, KS *p* = 1.31 × 10^−5^), Rigidity (Fisher’s *p* = 7.10 × 10^−10^, KS *p* = 1.45 × 10^−4^), Tremor (Fisher’s *p* = 9.35 × 10^−14^, KS *p* = 5.98 × 10^−6^), Dyskinesia (Fisher’s *p* = 9.85 × 10^−6^, KS *p* = 1.58 × 10^−4^), and Overall Severity (Fisher’s *p* = 1.86 × 10^−18^, KS *p* = 4.89 × 10^−6^). While Sialorrhea and Akathisia also achieved nominal significance under Fisher’s test (Fisher’s *p* = 6.66 × 10^−4^ and *p* = 4.76 × 10^−3^, respectively), neither reached significance in the KS test (KS *p* = 0.050 and *p* = 0.130), and Dystonia remained non-significant across both metrics (Fisher’s *p* = 0.286, KS *p* = 0.242). This divergence indicates that while primary motor signs distinguish the disease state from healthy physiology across the entire cohort, within-group acoustic heterogeneity in DIP is selectively sensitive to dyskinesia.

### 3.4. Machine Learning Results

#### 3.4.1. Binary DIP vs. HC Model

To evaluate the clinical diagnostic capability of the extracted multi-vowel nonlinear feature space, binary classification models were trained using eXtreme Gradient Boosting (XGBoost) and Random Forest (RF) algorithms. Model evaluation was conducted using 5-fold Group Cross-Validation grouped strictly by subject to ensure complete isolation across splits, with performance metrics computed directly on concatenated Out-of-Fold (OOF) predictions.

As summarized in Table 3, Random Forest achieved superior overall classification performance compared to XGBoost (McNemar’s test: *χ*^2^ = 4.08, *p* = 0.0433), yielding an Out-of-Fold ROC-AUC of 0.850 [0.759–0.922], an accuracy of 85.7% [78.6–92.0%], a *F*_1_-score of 0.91, and clinical sensitivity of 98.8% [96.2–100.0%]. XGBoost yielded a lower ROC-AUC of 0.800 [0.697–0.890] and an accuracy of 78.6% [70.5–85.7%]. The relative advantage of Random Forest may partly reflect its bagging architecture, which can reduce variance in modest-sized datasets. (*N*_DIP_ = 82, *N*_HC_ = 30). Notably, both models exhibited a specificity of 0.50 ([32.0–68.0%], [31.2–68.8%], respectively), indicating high sensitivity but limited specificity, likely reflecting the trade-off induced by class imbalance (*N*_DIP_: *N*_HC_ ≈ 2.73:1) prior to probability thresholding.

**Figure 7:**
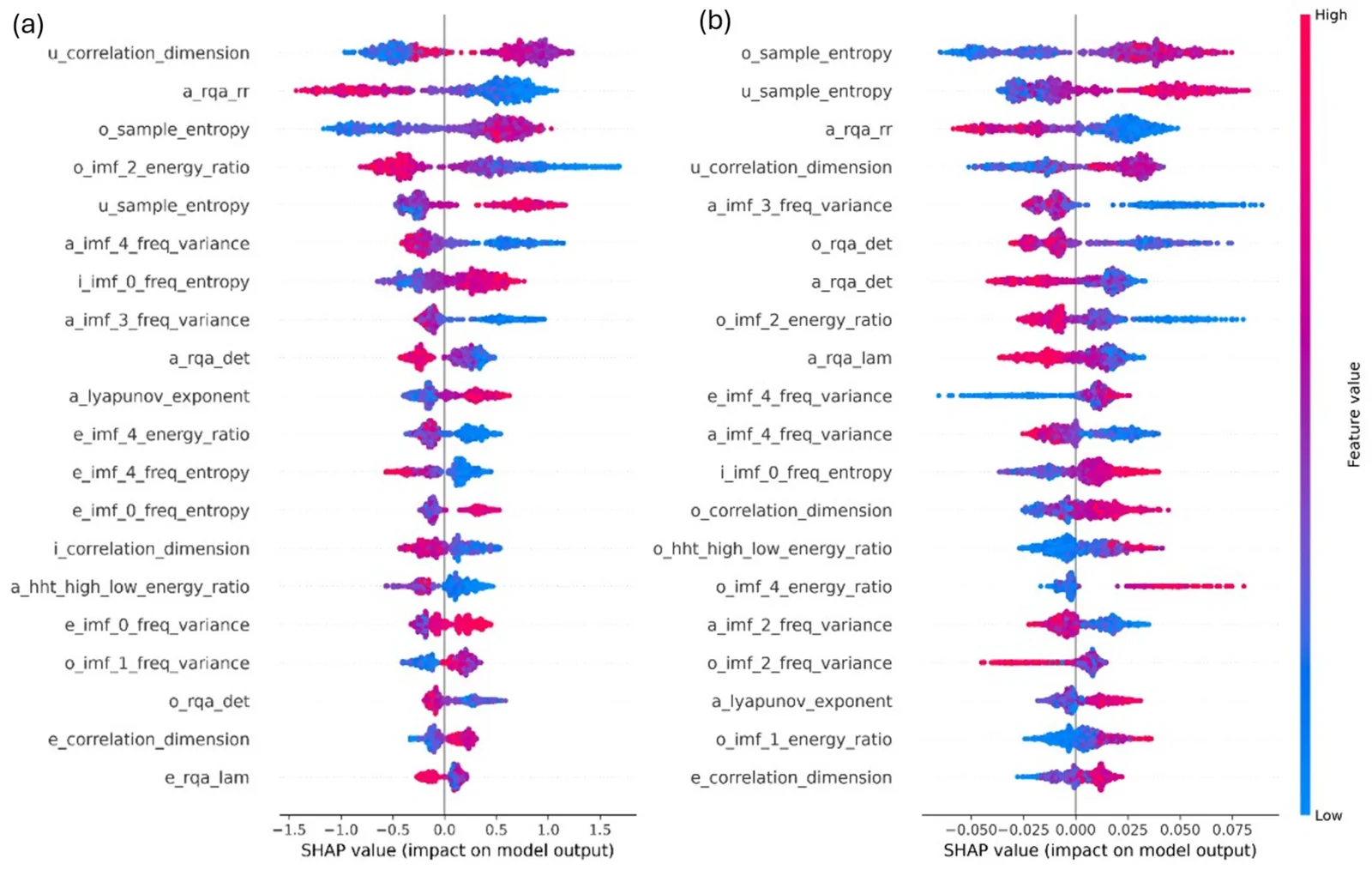
Shapley Summary Plot of DIP vs. HC Binary Classification: (**a**) XGBoost Model; (**b**) Random Forest Model.

To examine the features contributing to classifier predictions, Shapley Additive Explanations (SHAP) were computed across Out-of-Fold validation sets for both models, illustrated in Figure 7.

While nonlinear chaos metrics dominated predictive importance, specific HHT-derived features, such as o_imf_2_energy_ratio and a_imf_3_freq_variance, provided complementary diagnostic information, consistent with the statistical analyses and supporting HHT-derived measures as vowel-dependent augmentation features.

#### 3.4.2. Binary IPD vs. HC Model

To validate the generalizability and diagnostic efficacy of our physics-grounded nonlinear acoustic framework, a single-vowel (/a/) binary classification model was trained on the external Idiopathic Parkinson’s Disease cohort (*N*_IPD_ = 560, *N*_HC_ = 574). Benchmarking on IPD enables direct comparison with the extensive existing voice diagnostic literature. Model evaluation was executed via 5-fold cross-validation, with performance metrics calculated directly on out-of-fold (OOF) predictions.

**Figure 8:**
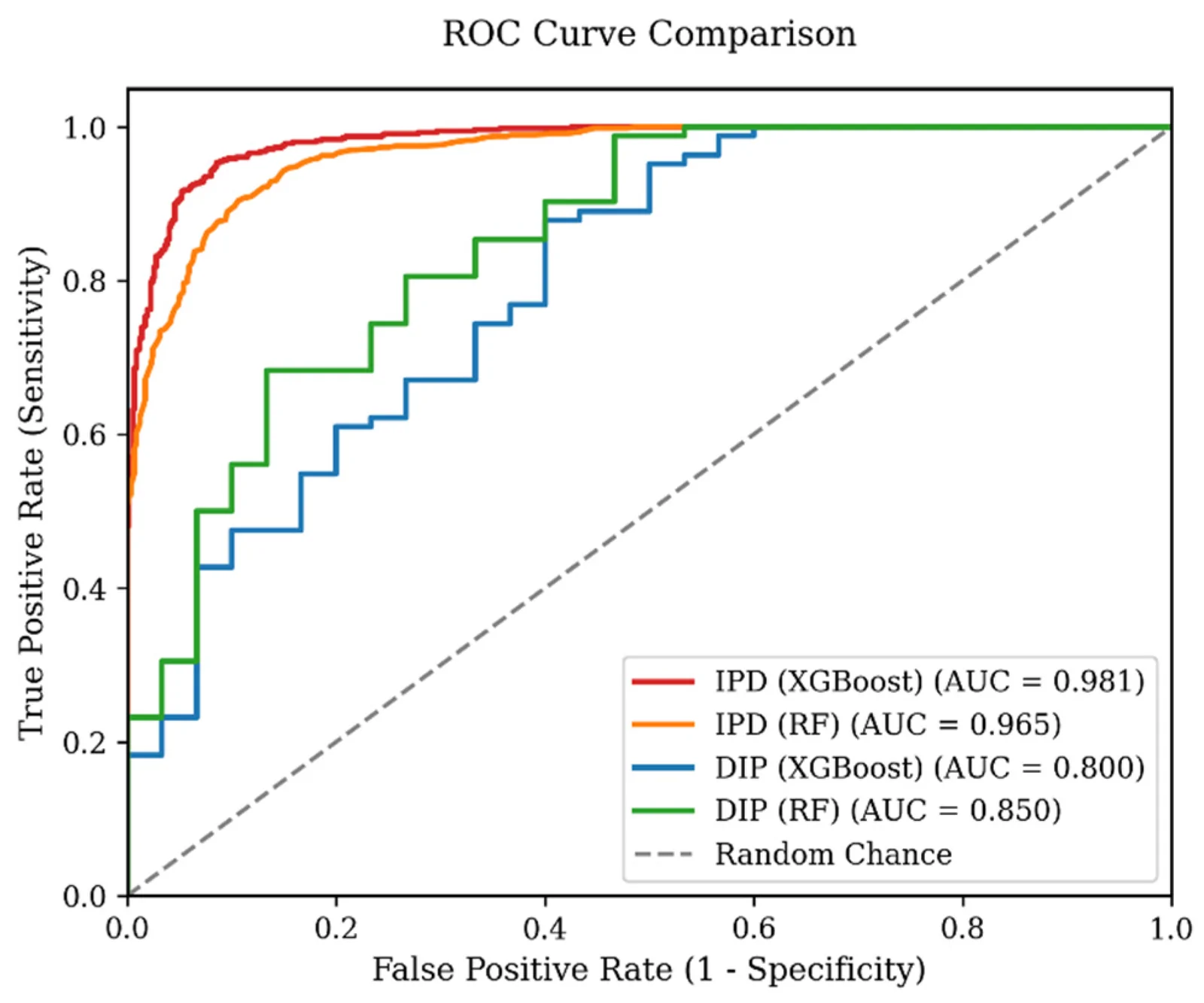
ROC-AUC Curve Comparisons in IPD-HC and DIP-HC Binary Classification.

As summarized in Table 4, XGBoost achieved better diagnostic performance compared to Random Forest (McNemar’s test: *χ*^2^ = 19.94, *p* < 0.0001), reaching an Out-of-Fold ROC-AUC of 0.981 [0.975–0.987], an accuracy of 93.0% [91.5–94.4%], and balanced sensitivity and specificity of 93.4% [91.4–95.4%] and 92.5% [90.3–94.6%]. Random Forest similarly yielded high performance, achieving an ROC-AUC of 0.965 [0.957–0.974] and an accuracy of 89.7% [88.0–91.4%]. The Receiver Operating Characteristic (ROC) curves across datasets and architectures are illustrated in Figure 8, highlighting the stark performance advantage achieved on the larger IPD cohort.

The 93% accuracy achieved by XGBoost aligns with median benchmark results reported across comprehensive systematic reviews of machine learning voice diagnostics for Parkinson’s disease [23]. Crucially, this suggests that replacing black-box feature extraction with interpretable HHT spectral parameters and nonlinear dynamic metrics incurs little-to-no performance trade-off, offering a potentially interpretable framework for neurogenic voice classification.

#### 3.4.3. Multiclass Ensemble Model

To achieve a differential diagnostic capability across all three clinical states, Drug-Induced Parkinsonism (DIP), Idiopathic Parkinson’s Disease (IPD), and Healthy Controls (HC), a multiclass classification architecture was evaluated. To address severe dataset size disparities across cohorts, a 7-Model Balanced Under-Sampling Ensemble Random Forest Model was deployed, enforcing a balanced 1:1:1 training ratio (*N* = 82 per class) per fold, with final Out-of-Fold (OOF) predictions aggregated via majority voting.

**Figure 9:**
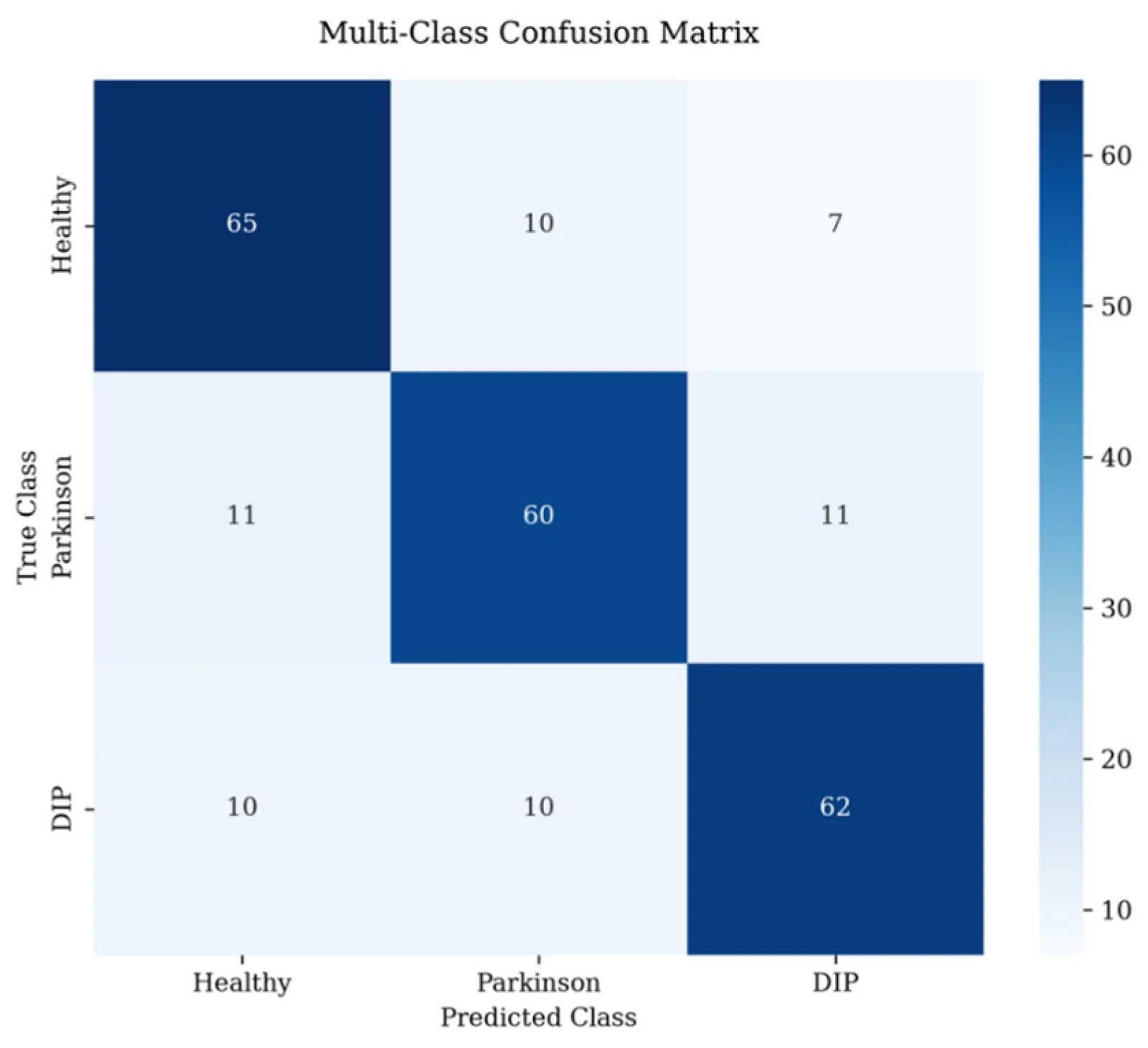
Confusion Matrix of Multiclass DIP-IPD-HC Classification.

As illustrated in Figure 9, the multiclass ensemble achieved an overall diagnostic accuracy of 76.0% (95% CI: 70.3–81.3%), with perfectly balanced macro-averaged precision of 0.760 (95% CI: 0.707–0.817), recall of 0.760 (95% CI: 0.703–0.813), and *F*_1_-score of 0.760 (95% CI: 0.703–0.813), alongside an overall macro One-vs-Rest ROC-AUC of 0.876 (95% CI: 0.837–0.912). Class-specific evaluation confirmed robust diagnostic stability across all three cohorts: the model identified 62 out of 82 DIP patients (Recall = 0.756, 95% CI: 0.659–0.854; Precision = 0.775, 95% CI: 0.697–0.859; *F*_1_-score = 0.765, 95% CI: 0.695–0.833; ROC-AUC = 0.902, 95% CI: 0.857–0.941); 65 out of 82 Healthy Controls (Recall = 0.793, 95% CI: 0.707–0.878; Precision = 0.756, 95% CI: 0.681–0.833; *F*_1_-score = 0.774, 95% CI: 0.706–0.836; ROC-AUC = 0.868, 95% CI: 0.814–0.917); and 60 out of 82 IPD patients (Recall = 0.732, 95% CI: 0.634–0.829; Precision = 0.750, 95% CI: 0.671–0.836; *F*_1_-score = 0.741, 95% CI: 0.663–0.812; ROC-AUC = 0.859, 95% CI: 0.801–0.910). Cross-pathology confusions occurred symmetrically between IPD and DIP, with 11 IPD instances misclassified as DIP and 11 DIP instances misclassified as IPD.

The balanced diagonal performance across all three classes confirms that the 7-model under-sampling ensemble successfully prevented predictive bias toward any single cohort. Crucially, the model demonstrates that nonlinear chaos dynamics and Hilbert–Huang spectral metrics contain sufficient discriminative power to separate not only healthy voices from pathological speech, but also to differentiate Drug-Induced Parkinsonism from Idiopathic Parkinson’s Disease.

## 4. Discussion

The findings of this study establish that physics-grounded nonlinear acoustics and Hilbert–Huang spectral metrics provide a sensitive, non-invasive probe for detecting and differentiating Drug-Induced Parkinsonism (DIP) from Idiopathic Parkinson’s Disease (IPD) and healthy states. By shifting the acoustic analytical paradigm from traditional linear perturbation measures to phase-space chaos and empirical mode decomposition, this framework addresses the fundamental physical reality of human phonation as a nonlinear, non-stationary dynamical system. The marked elevation in Sample Entropy and Correlation Dimension (*D*_2_) observed in DIP patients reflects an increase in active degrees of freedom and chaotic trajectory divergence during vocal-fold oscillation. Biomechanically, antipsychotic-induced blockade of central dopamine receptors induces extrapyramidal hypertonicity, muscle rigidity, and incomplete glottal closure. When subglottal airflow passes through an irregularly approximated glottis, smooth periodic oscillation degenerates into turbulent, aperiodic tissue vibration, driving the system toward higher-dimensional chaos [39].

HHT-derived frequency variability exhibited clear vowel-dependent behavior. For example, during sustained /a/ phonation, frequency variance was reduced in the lower-frequency IMFs (IMF 4, IMF 3, and IMF 2), whereas sustained /e/ and /u/ phonations showed increased variance in IMF 4 and IMF 1, respectively. Although reduced fundamental frequency variability during connected or running speech and increased jitter during sustained phonation have been reported in Parkinson’s disease in specific study contexts [9,12,40], our findings in DIP suggest that frequency variability is not expressed uniformly across phonetic conditions. This interpretation is consistent with evidence that fundamental frequency and related acoustic variability are influenced by speech-task and linguistic characteristics, including lexical stress and differences between sustained phonation and connected speech [41,42,43]. Accordingly, HHT-derived frequency variability may be better interpreted as a phoneme- and task-sensitive marker rather than a universal disease signature.

In addition, our findings reveal a second form of phoneme-specific sensitivity related to articulatory geometry. The heightened nonlinearity observed in the high-to-mid back rounded vowels /u/ and /o/ is consistent with source–filter acoustic coupling. Lip rounding and posterior tongue elevation constrict the anterior vocal tract and lengthen the acoustic tube, creating a semi-occluded vocal tract (SOVT) configuration [36,37,38]. In healthy phonation, the resulting supraglottal backpressure and inertive reactance may contribute to more stable vocal-fold oscillation. Under extrapyramidal motor dysfunction, however, this acoustic loading may make underlying glottal irregularities more acoustically apparent. This may help explain why /u/ and /o/ showed the largest Sample Entropy effect sizes (d = +0.95 and d = +0.80, respectively).

While entropy metrics showed vowel-dependent significance, RQA demonstrated more consistent cross-vowel abnormalities, particularly for determinism (DET), which was significantly reduced across all five vowels. This pattern is consistent with previous voice-disorder studies showing reduced diagonal recurrence structures and lower regularity in pathological phonation, with DET also reported to be lower in disordered than in normal voices [44,45]. In contrast, recurrence rate (RR) and laminarity (LAM) also tended to decrease across vowels in our DIP cohort, but their statistical significance was less consistent. Notably, prior studies have not shown uniform changes in these measures; for example, LAM may remain unchanged or even increase slightly in some pathological voice datasets. Such discrepancies may reflect differences in the underlying disorder, recording protocol, embedding parameters, recurrence threshold, or RQA implementation. Overall, these findings suggest that reduced DET may represent a relatively robust marker of disrupted vocal dynamical regularity, whereas RR and LAM may be more sensitive to disease- and method-specific conditions.

Analysis within the DIP cohort using individual DIEPSS domains demonstrated that acoustic abnormalities were most prominently associated with dyskinesia compared with the other extrapyramidal symptom dimensions. Several findings may explain this selective association. First, dyskinesia has been reported to demonstrate the highest node-strength centrality within the extrapyramidal symptom network, suggesting that it represents a highly interconnected domain of antipsychotic-induced motor dysfunction [46]. Second, patients with dyskinesia have demonstrated substantially greater segment-to-segment fluctuations in formant frequencies [47], consistent with unstable articulatory motor control. Related studies have also described excessive formant fluctuations and vowel distortion during sustained phonation in dysarthric speech [48]. Clinically, dyskinetic movements may involve oral and pharyngeal musculature as well as other body regions; involvement of the mouth and throat can interfere directly with articulation and may coexist with salivation, swallowing difficulty, reduced speech rate, and impaired pronunciation [49,50]. Collectively, these observations provide a plausible clinical and biomechanical basis for the particularly strong association between dyskinesia and the nonlinear acoustic abnormalities observed within our DIP cohort.

When healthy controls, who by definition had no extrapyramidal manifestations and were therefore assigned a score of zero across DIEPSS domains, were included in the analysis, a broader pattern emerged. Gait, bradykinesia, rigidity, tremor, dyskinesia, and overall severity all showed significant acoustic associations. This broader involvement of parkinsonian motor domains in vocal dysfunction is also supported by findings from Parkinson’s disease, as evidenced by several clinical and acoustic observations. Re-emergent vocal tremors during sustained vowel phonation have been reported in a patient who also exhibited rigidity and mild bradykinesia during longitudinal follow-up [51]. Moreover, although vocal tremor was acoustically detectable at similar frequencies in both patients with Parkinson’s disease and healthy controls, patients with Parkinson’s disease showed significantly greater tremor amplitude [52]. Smoothed cepstral peak prominence (CPPS) has also shown trends toward associations with rigidity and bradykinesia [53]. These findings are consistent with the clinical characteristics of hypokinetic dysarthria, in which rigidity and bradykinesia contribute to articulatory undershoot, breathy phonation, and reduced vocal intensity [54]. Moreover, the contribution of overall severity is supported by studies showing that higher Hoehn–Yahr stages and Unified Parkinson’s Disease Rating Scale (UPDRS) scores are associated with greater perceptual voice impairment, reduced phonatory performance, and poorer speech articulation [55,56,57], suggesting that vocal abnormalities may reflect the cumulative burden of parkinsonian motor dysfunction.

Taken together, these findings demonstrate substantial clinical and acoustic overlaps between DIP and PD, suggesting broadly convergent effects of parkinsonian motor dysfunction on phonatory and articulatory control. Dyskinesia, however, may represent an important point of divergence. Unlike bradykinesia, rigidity, tremor, and gait disturbance, dyskinesia is not a cardinal manifestation of untreated PD and is most commonly recognized as a treatment-related motor complication, particularly following chronic levodopa exposure [58,59,60]. In contrast, within our DIP cohort, dyskinesia emerged as the DIEPSS domain most strongly associated with acoustic abnormalities. This finding suggests that dyskinesia-related vocal instability may represent a distinctive feature of DIP beyond the acoustic manifestations shared with PD.

Exploratory comparison between the DIP and IPD cohorts identified distinct normalized nonlinear acoustic profiles. Relative to their respective healthy-control reference groups, the DIP cohort showed greater reductions in phase-space determinism, laminarity, and recurrence rate, together with higher Sample Entropy and Correlation Dimension than the IPD cohort. These differences are compatible with the distinct clinical and pathophysiological contexts of the two disorders; however, given the cross-dataset design, they should not be interpreted as direct evidence of disease-specific neurobiological mechanisms. IPD is a progressive neurodegenerative disorder that frequently presents with asymmetric motor involvement, whereas DIP arises in the context of pharmacological dopamine-receptor blockades and is often characterized by more symmetric motor manifestations. Whether these differences contribute to the observed patterns of laryngeal instability and phase-space dynamics warrants investigation in prospectively matched cohorts recorded under standardized conditions. As a proof-of-concept analysis, the balanced multiclass ensemble achieved an overall accuracy of 76.0%, with comparable class-specific F_1_-scores across DIP, IPD, and healthy controls. These findings suggest that nonlinear voice features may contain information relevant to differentiating drug-induced from idiopathic parkinsonism, while prospective external validation is required before clinical diagnostic application.

Despite these promising findings, several methodological limitations should be acknowledged. First, the primary DIP cohort was relatively small (*N*_DIP_ = 82, *N*_HC_ = 30), which may have contributed to the limited specificity observed in binary classification. More importantly, all participants in the DIP cohort had schizophrenia and were receiving antipsychotic treatment, whereas the healthy controls had neither schizophrenia nor antipsychotic exposure. The present design therefore cannot fully disentangle acoustic alterations attributable to DIP from those associated with schizophrenia itself or antipsychotic exposure independent of extrapyramidal motor effects. Previous studies have demonstrated acoustic abnormalities in schizophrenia, particularly reduced pitch variability and alterations in speech timing, including pauses and the proportion of spoken time; however, these effects are heterogeneous across studies, languages, and speech tasks, and a large cross-linguistic investigation identified only a limited generalizable acoustic profile of schizophrenia [61]. Within our DIP cohort, however, the observed associations between DIEPSS motor domains and acoustic measures provide complementary evidence that extrapyramidal motor dysfunction contributes to the phonatory abnormalities observed in this population. In particular, dyskinesia showed the strongest within-cohort enrichment of acoustic associations, while gait disturbance, bradykinesia, rigidity, tremor, and overall extrapyramidal severity collectively support a broader relationship between motor dysfunction and phonation. Nevertheless, these within-cohort associations do not establish that the between-group acoustic differences are specific to DIP. Future studies should therefore include an antipsychotic-treated schizophrenia control group without DIP or clinically significant extrapyramidal symptoms, ideally together with antipsychotic-naïve or minimally exposed psychiatric controls, to distinguish disease-, medication-, and motor-related acoustic effects.

Age also differed substantially between the DIP and healthy-control groups and represents an additional potential confounder. To address this issue, age- and sex-adjusted regression analyses with Benjamini–Hochberg false discovery rate correction were performed for the prespecified nonlinear acoustic features. Fifteen of the 20 vowel–feature associations remained significant after demographic adjustment and FDR correction, with the most robust effects involving recurrence quantification measures and Sample Entropy (Appendix A). These sensitivity analyses indicate that the principal nonlinear findings cannot be explained solely by the observed age difference, although residual demographic confounding remains possible. Future studies should recruit larger, age- and sex-matched cohorts across diverse psychiatric settings to further establish generalizability.

Second, cross-disease comparisons combined our primary multi-vowel dataset with an external IPD dataset containing sustained /a/ phonation. Although Z-score normalization relative to the corresponding healthy-control cohort was applied to reduce dataset-related scale differences, this procedure cannot eliminate batch effects arising from differences in microphone hardware, sampling characteristics, recording environments, or recruitment procedures. Accordingly, the DIP–IPD comparisons and multiclass classification should be regarded as exploratory cross-dataset analyses rather than external validation of a disease-specific diagnostic classifier.

Third, the cross-cohort comparison between DIP and IPD could not account directly for matched motor severity because comparable clinical rating scales were not available across datasets. Although DIEPSS scores were available for the DIP cohort, equivalent standardized motor assessments were unavailable for the external IPD cohort. Differences in nonlinear acoustic dynamics between DIP and IPD therefore cannot be attributed solely to differences in underlying pathophysiology. Future prospective studies should acquire standardized multi-vowel recordings together with harmonized motor assessments in matched DIP, IPD, antipsychotic-treated schizophrenia without DIP, and healthy-control cohorts under identical recording conditions. Such a design would enable direct evaluation of disease-specific acoustic signatures, adjustment for motor severity and medication exposure, and more rigorous validation of voice-based differential classification.

## 5. Conclusions

This study establishes a physics-grounded, nonlinear acoustic framework for characterizing laryngeal motor dysfunction in Drug-Induced Parkinsonism (DIP). By integrating interpretable Hilbert–Huang Transform (HHT)-derived spectral descriptors with entropy, phase-space chaos, and Recurrence Quantification Analysis (RQA), the framework extends conventional acoustic analysis beyond purely discriminative features toward a more mechanistically interpretable description of vocal dynamics. HHT-derived parameters provided vowel-dependent information on frequency variability and energy organization, whereas nonlinear measures revealed greater vocal irregularity and attractor complexity in DIP. Most notably, RQA demonstrated a consistent reduction in recurrence rate, determinism, and laminarity across phonemic contexts, indicating a generalized loss of stable and recurrent vocal-fold dynamics.

The multi-vowel analysis further showed that rounded back vowels, particularly /u/ and /o/, were especially sensitive to nonlinear abnormalities, supporting an interaction between laryngeal instability and vowel-dependent source–filter coupling. Within the DIP cohort, DIEPSS analysis identified dyskinesia as the most prominent symptom-specific acoustic correlate. In contrast, when associations were examined across healthy controls and patients with DIP, a broader pattern emerged, with gait disturbance, bradykinesia, rigidity, tremor, dyskinesia, and overall severity all showing significant acoustic associations. These findings suggest that, at the group level, DIP-related vocal abnormalities reflect the convergent effects of multiple parkinsonian motor disturbances on phonatory and articulatory control, whereas dyskinesia may represent a more distinctive source of inter-individual acoustic variability within DIP.

Finally, in exploratory classification analyses, these interpretable acoustic features demonstrated discriminative potential. The Random Forest model achieved a ROC-AUC of 0.85, with 99% sensitivity and 50% specificity for distinguishing DIP from healthy controls, while the balanced three-class ensemble achieved 76% accuracy in differentiating DIP, IPD, and healthy participants. Although the binary RF classifier showed high sensitivity, its limited specificity indicates an asymmetric classification profile, with strong DIP case detection accompanied by a substantial false-positive rate among healthy controls. Therefore, the high sensitivity should not be interpreted as evidence of clinical diagnostic readiness. Rather, these findings should be considered proof-of-concept evidence that nonlinear vocal features contain discriminative information associated with the DIP phenotype. Further prospective validation in independent and clinically relevant comparison cohorts is required to establish their diagnostic utility.

Taken together, these findings support nonlinear voice analysis as a non-invasive and interpretable approach for characterizing laryngeal motor instability and symptom-related extrapyramidal effects, while highlighting its potential role in the future differentiation of drug-induced and idiopathic parkinsonism.

## Figures and Tables

**Table 1:** Vowel Characterization and DIP-Control Entropy Statistical Comparison.

Vowel	Tongue Position	Lip Shape	Entropy Cohen’s D	*p*
U	High-Back	Rounded	+0.96	3.06 × 10^−5^
O	Mid-Back	Rounded	+0.80	2.67 × 10^−6^
E	Mid-Front	Unrounded	+0.47	0.04
A	Low-Front	Unrounded	+0.33	0.25
I	High-Front	Unrounded	+0.30	0.32

**Table 2:** Comparison of HC-referenced standardized nonlinear acoustic features between DIP and IPD, adjusted with permutation testing and false discovery rates.

Nonlinear Feature	DIP Z	IPD Z	Mann–Whitney *p*	*p_perm_*	*FDR_perm_*	Cohen’s d
RQA RR	−0.81	0.16	9.53 × 10^−12^	<1.00 × 10^−4^	4.40 × 10^−4^	−0.61
RQA DET	−0.98	−0.03	7.13 × 10^−10^	<1.00 × 10^−4^	4.40 × 10^−4^	−0.73
RQA LAM	−0.97	−0.17	1.12 × 10^−6^	<1.00 × 10^−4^	4.40 × 10^−4^	−0.58
Correlation Dimension	0.36	0.00	5.60 × 10^−4^	9.60 × 10^−3^	2.35 × 10^−2^	0.31
Sample Entropy	0.47	−0.17	7.30 × 10^−4^	<1.00 × 10^−4^	4.40 × 10^−4^	0.52
Lyapunov Exponent	0.34	0.02	1.45 × 10^−2^	2.71 × 10^−2^	5.42 × 10^−2^	0.27

**Table 3:** Out-of-Fold performance metrics for binary classification between Drug-Induced Parkinsonism (DIP) and Healthy Controls (HC). (95% CI.)

Model	AUC	Accuracy	Sensitivity	Specificity	F1-Score
Random Forest	0.850 [0.759–0.922]	85.7% [78.6–92.0%]	98.8% [96.2–100.0%]	50.0% [32.0–68.0%]	0.91
XGBoost	0.800 [0.697–0.890]	78.6% [70.5–85.7%]	89.0% [81.9–95.2%]	50.0% [31.2–68.8%]	0.86

**Table 4:** Out-of-Fold performance metrics for binary classification between Idiopathic Parkinson’s Disease (IPD) and Healthy Controls (HC). (95% CI.)

Model	AUC	Accuracy	Sensitivity	Specificity	F1-Score
XGBoost	0.981 [0.975–0.987]	93.0% [91.5–94.4%]	93.4% [91.4–95.4%]	92.5% [90.3–94.6%]	0.93
Random Forest	0.965 [0.957–0.974]	89.7% [88.0–91.4%]	89.3% [86.4–91.7%]	90.1% [87.5–92.4%]	0.90

**Figure A1:**
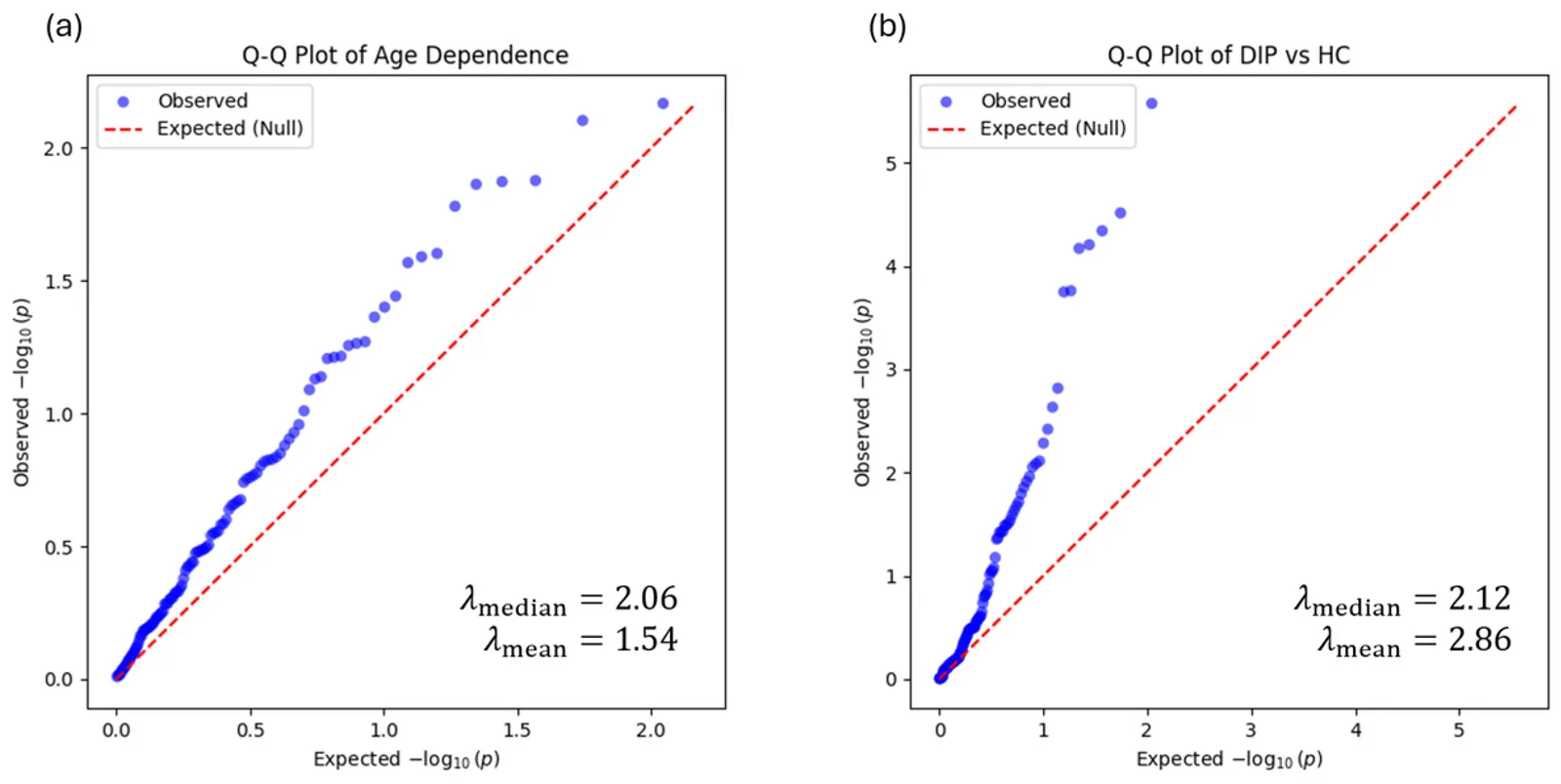
Quantile–Quantile plot of (**a**) logarithmic *p*-value distribution of Spearman age correlation, Median Inflation Factor *λ_median_* = 2.06, Mean Inflation Factor *λ_mean_* = 1.54, KS *p*-value 2.20 × 10^−3^ (**b**) logarithmic *p*-value distribution of DIP vs. HC significance, Median Inflation Factor *λ_median_* = 2.12, Mean Inflation Factor *λ_mean_* = 2.86, and KS *p*-value 5.89 × 10^−6^.

**Figure A2:**
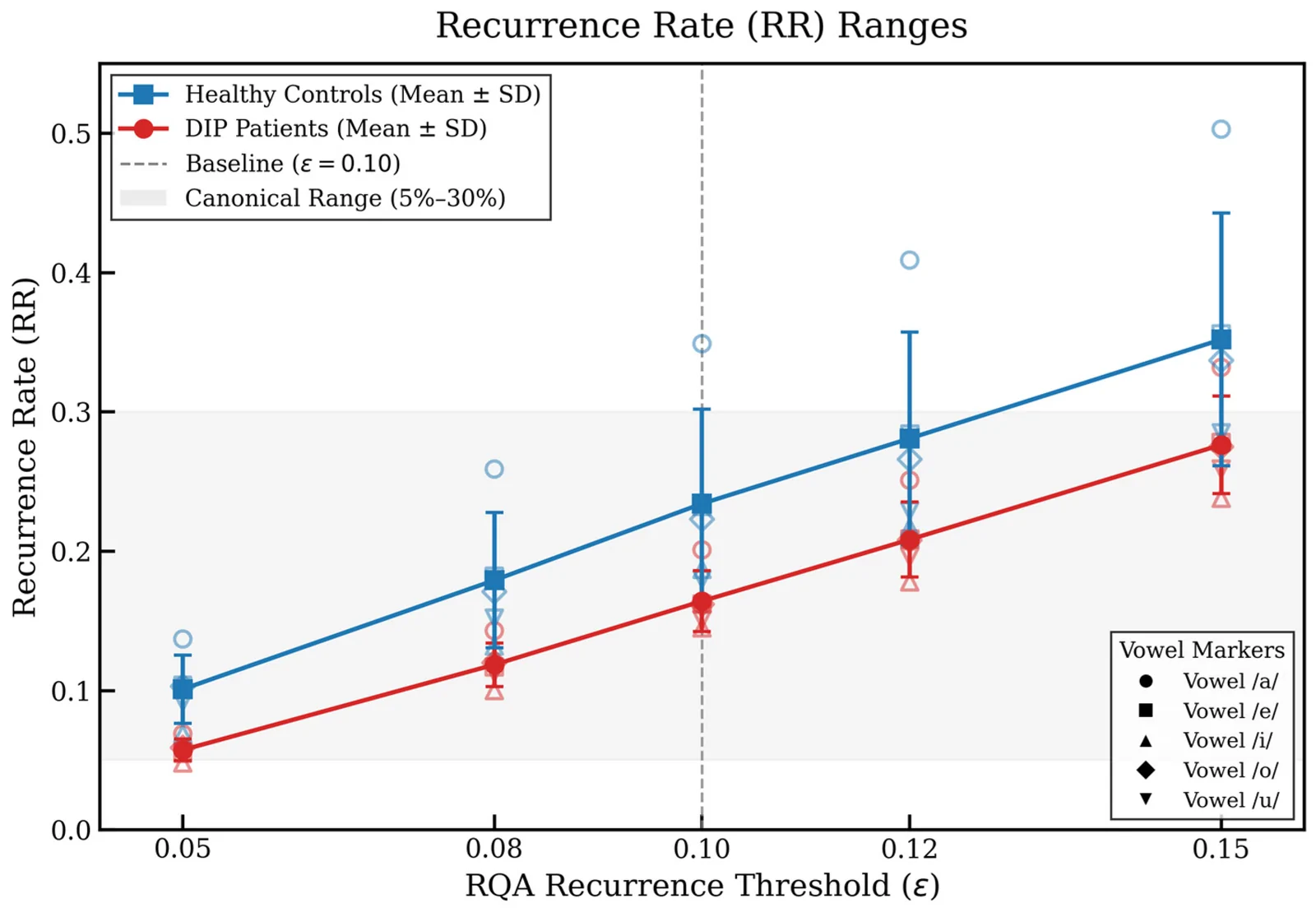
Empirical Recurrence Rate (RR) scaling across varying recurrence thresholds (ϵ) for five cardinal vowels in Drug-Induced Parkinsonism (DIP) and Healthy Controls (HC).

**Figure A3:**
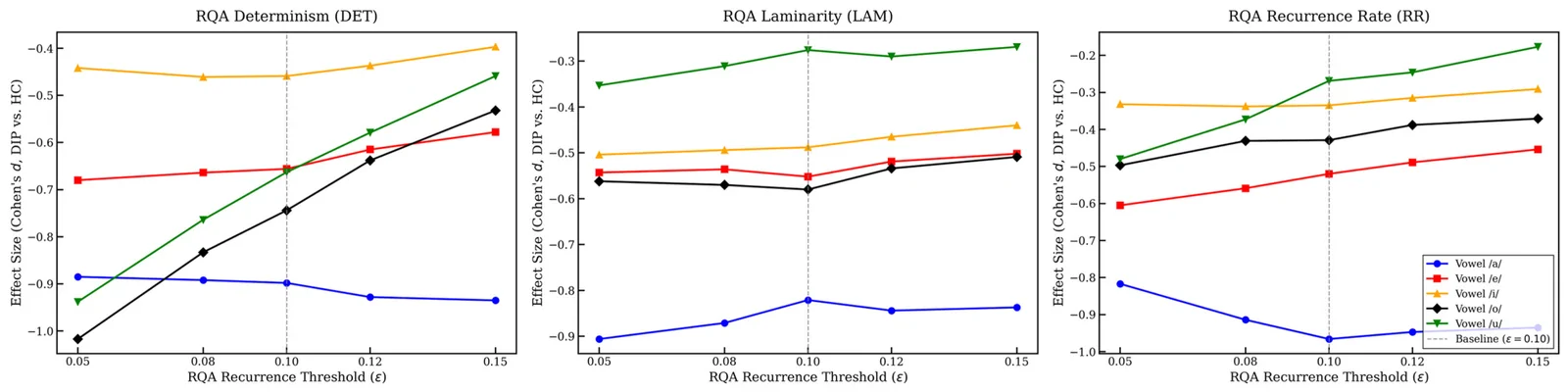
Cohen’s d Effect Size of RQA Determinism (DET), Laminarity (LAM), and Recurrence Rate (RR) across *ϵ* ∈ [0.05, 0.15].

## Data Availability

The raw data supporting the conclusions of this article will be made available by the authors on request.

## Supplementary Materials

The following supporting information can be downloaded at: https://www.mdpi.com/article/10.3390/diagnostics16193187/s1. File S1: Age_Correlation_Analysis.xlsx, File S2: DIEPSS_Correlation_Results_Full_Cohort.xlsx, File S3: DIEPSS_Correlation_Results_Within_DIP_Cohort.xlsx, File S4: DIP vs HC statistical results.csv, File S5: DIP vs IPD statistical results.csv, File S6: IPD vs HC statistical results.csv.

## Abbreviations

The following abbreviations are used in this manuscript:

DIP	Drug-Induced Parkinsonism
EPS	Extrapyramidal Symptoms
DIEPSS	Drug-Induced Extrapyramidal Symptoms Scale
ROC	Receiver Operating Characteristic
AUC	Area Under the Curve
IPD	Idiopathic Parkinson’s Disease
HC	Healthy Control
HHT	Hilbert–Huang Transform
IMF	Intrinsic Mode Function
EEMD	Ensemble Empirical Mode Decomposition
RQA	Recurrence Quantification Analysis
RR	Recurrence Rate
DET	Determinism
LAM	Laminarity
FDR	False Discovery Rate
RF	Random Forest
XGBoost	eXtreme Gradient Boosting
OOF	Out-of-Fold
SOVT	Semi-occluded Vocal Tract
SHAP	Shapley Additive Explanations
KS	Kolmogorov–Smirnov

## Appendix A

### Appendix A.1. Age-Related Associations

Analysis of age via Spearman rank correlations verifies the validity of our findings. An overall feature inflation factor of *λ_median_* = 2.06 was obtained by the methods mentioned above. Though the Kolmogorov–Smirnov (KS) Test (*p* = 2.20 × 10^−3^) indicates a significant difference, further discussion into confounding factors could explain different mechanisms compared to DIP-HC, thus supporting that the aforementioned results in the paper are not artifacts of inadequate age control (F = 49.7, *p* < 0.01).

This point could be made due to the following reasons. First, when assessing KS group significance with a uniform distribution, results from DIP vs. HC are more significant compared to age group analysis, with KS statistics of 0.237 and 0.174, respectively, as visualized in QQ plots in Figure A1. This means that DIP contributes more changes in physics biomarkers to what pure age provides. Second, as detailed in Table A1, features of significance in Spearman age analysis do not overlap with DIP vs. HC. Third, supplementary findings indicate that age alters vowel-specific HHT results like IMF 4 energy ratio (/o/) or IMF 2 frequency entropy (/e/), while DIP alters broad nonlinear metrics like sample entropy, correlation dimension, and RQA results, pointing to mechanistic differences.

**Table A1:** Features of significant correlation by age within DIP cohort.

	Age Spearman		DIP vs. HC	
Nonlinear Feature	Correlation	*p*	Cohen’s d	*p*
o_imf_4_energy_ratio	0.298488	0.006797	0.310231	0.775026
e_imf_3_energy_ratio	0.29329	0.007877	−0.12031	0.316359
e_imf_2_freq_entropy	0.27416	0.013259	−0.15636	0.676523
u_imf_3_freq_variance	0.273798	0.013386	0.076922	0.681335
o_imf_2_freq_variance	0.272928	0.013695	−0.38156	0.090675
e_imf_2_freq_variance	0.265572	0.016566	0.026859	0.83091
o_imf_3_freq_entropy	−0.24906	0.024949	−0.18896	0.652664
o_imf_3_energy_ratio	0.247877	0.02567	−0.02682	0.488206
o_imf_3_freq_variance	0.245742	0.027013	−0.01268	0.934545
o_imf_1_energy_ratio	−0.23326	0.036111	0.521977	0.023206
o_lyapunov_exponent	0.22903	0.03972	−0.11588	0.705586
i_imf_1_freq_entropy	0.225131	0.04331	−0.22529	0.265424

### Appendix A.2. Adjusted Regression of Main DIP vs. HC Features

To address potential demographic confounding, multivariable regression models were fitted for the prespecified primary nonlinear acoustic features, with diagnostic group, age, and sex entered simultaneously as predictors. Benjamini–Hochberg false discovery rate correction was applied across the 20 primary vowel–feature comparisons.

After adjustment for age and sex, 15 of the 20 prespecified nonlinear vowel–feature associations remained significant after FDR correction. The most robust findings involved RQA metrics, particularly recurrence rate, determinism, and laminarity, together with increased Sample Entropy in rounded back vowels /u/, /o/, and /e/. These findings indicate that the principal DIP-associated nonlinear acoustic abnormalities cannot be explained solely by the substantial age difference between the DIP and healthy-control cohorts.

**Table A2:** Adjusted regression for DIP vs. HC nonlinear features, FDR-corrected.

Feature	Unadjusted *β*	Age + Sex Adjusted *β*	95% CI	*p*-Value	FDR *q*
a_rqa_rr	−0.915	−1.039	[−1.513, −0.564]	3.25 × 10^−5^	6.55 × 10^−4^
a_rqa_det	−0.838	−1.020	[−1.500, −0.540]	5.24 × 10^−5^	6.55 × 10^−4^
a_rqa_lam	−0.765	−0.957	[−1.442, −0.472]	1.63 × 10^−4^	0.0014
e_rqa_lam	−0.522	−0.898	[−1.386, −0.410]	4.13 × 10^−4^	0.0024
u_sample_entropy	+0.882	+0.870	[0.391, 1.349]	4.82 × 10^−4^	0.0024
o_rqa_lam	−0.571	−0.868	[−1.357, −0.380]	6.27 × 10^−4^	0.0025
o_rqa_det	−0.728	−0.861	[−1.350, −0.372]	7.05 × 10^−4^	0.0025
u_rqa_det	−0.681	−0.835	[−1.329, −0.340]	0.0011	0.0035
o_sample_entropy	+0.771	+0.810	[0.320, 1.301]	0.0014	0.0040
e_rqa_det	−0.624	−0.796	[−1.295, −0.298]	0.0020	0.0050
u_rqa_lam	−0.342	−0.672	[−1.151, −0.192]	0.0065	0.0148
a_correlation_dimension	+0.408	+0.634	[0.145, 1.123]	0.0115	0.0239
e_rqa_rr	−0.493	−0.644	[−1.148, −0.140]	0.0128	0.0246
i_rqa_lam	−0.540	−0.561	[−1.058, −0.065]	0.0271	0.0483
e_sample_entropy	+0.515	+0.566	[0.059, 1.073]	0.0291	0.0485
o_rqa_rr	−0.440	−0.531	[−1.038, −0.024]	0.0405	0.0598
i_sample_entropy	+0.408	+0.533	[0.023, 1.043]	0.0406	0.0598
i_rqa_det	−0.531	−0.501	[−1.008, 0.006]	0.0528	0.0733
u_rqa_rr	−0.301	−0.477	[−0.975, 0.021]	0.0601	0.0791
a_sample_entropy	+0.323	+0.453	[−0.034, 0.940]	0.0682	0.0852

## Appendix B

To ensure reproducible model development and minimize optimistic bias arising from hyperparameter selection, Random Forest (RF) and XGBoost classifiers were optimized using a nested cross-validation framework. The outer validation loop used four-fold Stratified Group K-Fold cross-validation, preserving class stratification while ensuring that observations originating from the same participant were assigned exclusively to either the training or validation fold. Hyperparameter optimization was conducted only within the training portion of each outer fold, thereby preventing information from the outer validation fold from influencing model selection.

### Appendix B.1. Random Forest in Binary Classification (DIP-HC, IPD-HC)

For the Random Forest classifier, hyperparameter optimization was performed using RandomizedSearchCV within each outer training fold. The inner optimization used three-fold cross-validation, with 30 randomly sampled hyperparameter combinations (n_iter = 30) and the area under the receiver operating characteristic curve (ROC-AUC) as the optimization criterion (scoring = ‘roc_auc’). Class imbalance was addressed by setting class_weight = ‘balanced’.

The hyperparameter search space was as follows.

**Table A3:** Hyperparameter search for Binary Random Forest.

Hyperparameter	Search Space
n_estimators	100, 200, 300, 500
max_depth	3, 5, 10, 15, None
min_samples_split	2, 5, 10
min_samples_leaf	1, 2, 4
max_features	sqrt, log2, None
bootstrap	True, False
class_weight	balanced
Optimization metric	ROC-AUC

Thus, model selection occurred exclusively within the inner cross-validation loop, whereas performance estimates were obtained from the held-out folds of the outer Stratified Group K-Fold procedure.

### Appendix B.2. XGBoost in Binary Classification (DIP-HC, IPD-HC)

The XGBoost classifier was evaluated using the same nested cross-validation principle. Hyperparameter optimization was restricted to the training data within each outer fold to avoid information leakage between model selection and performance evaluation. Class imbalance was additionally addressed using scale_pos_weight.

**Table A4:** Hyperparameter search for Binary XGBoost.

Hyperparameter	Search Space
n_estimators	100, 200, 300
learning_rate	0.01, 0.05, 0.10, 0.20
max_depth	3, 4, 5, 6, 7
subsample	0.6, 0.8, 1.0
colsample_bytree	0.6, 0.8, 1.0
min_child_weight	1, 3, 5
gamma	0, 0.1, 0.3, 0.5
reg_alpha	0, 0.1, 1, 10
reg_lambda	1, 1.5, 2, 5
scale_pos_weight	Set according to class imbalance
Optimization metric	ROC-AUC

### Appendix B.3. Multiclass Ensemble Models

To address the substantial class-size imbalance among the DIP, IPD, and HC cohorts, multiclass classification was implemented using a 7-model balanced under-sampling ensemble strategy. For each constituent model, the larger IPD and HC cohorts were randomly under-sampled to match the DIP cohort, yielding a balanced 1:1:1 sample distribution (*N* = 82 per class; total *N* = 246) within each balanced subset. Seven independently under-sampled constituent models were generated, and their multiclass predictions were aggregated by majority voting to obtain the final ensemble prediction.

This ensemble framework was implemented separately using Random Forest (RF) and XGBoost as the base classifier architectures. Hyperparameter optimization for each architecture was performed using RandomizedSearchCV with 30 randomly sampled parameter combinations. Because each constituent training subset was explicitly balanced across the three classes, multiclass accuracy was used as the optimization criterion.

#### Appendix B.3.1. Random Forest Under-Sampling Ensemble

For each constituent Random Forest classifier, hyperparameters were optimized over the following search space.

**Table A5:** Hyperparameter search for Ensemble Multiclass Random Forest.

Hyperparameter	Search Space
n_estimators	100, 200, 300, 500
max_depth	5, 10, 15, None
min_samples_split	2, 5, 10
min_samples_leaf	1, 2, 4
max_features	sqrt, log2
Optimization metric	Accuracy

The final 7-model Random Forest balanced under-sampling ensemble achieved an overall accuracy of 76.0% (187/246; 95% CI: 70.3–81.3%). Macro-averaged precision, recall, and F1-score were all approximately 0.76, indicating relatively balanced classification performance across the three diagnostic classes.

#### Appendix B.3.2. XGBoost Under-Sampling Ensemble

The same seven-model balanced under-sampling ensemble framework was independently implemented using XGBoost as the base classifier. Hyperparameters were optimized over the following search space.

**Table A6:** Hyperparameter search for Ensemble Multiclass XG Boost.

Hyperparameter	Search Space
n_estimators	100, 200, 300, 500
max_depth	3, 4, 5, 6, 8
learning_rate	0.01, 0.05, 0.10, 0.20
subsample	0.6, 0.8, 1.0
colsample_bytree	0.6, 0.8, 1.0
gamma	0, 0.1, 0.2, 0.5, 1.0
Randomized-search iterations	30
Optimization metric	Accuracy

The corresponding 7-model XGBoost balanced under-sampling ensemble achieved an overall accuracy of 66.3% (163/246; 95% CI: 60.6–72.0%). Macro-averaged precision, recall, and F1-score were 0.67, 0.66, and 0.67, respectively. DIP showed the highest class-specific precision (0.81) and F1-score (0.75).

#### Appendix B.3.3. Comparison of Ensemble Architectures

Among the two balanced under-sampling ensemble architectures, the Random Forest ensemble showed higher overall multiclass accuracy than the XGBoost ensemble (76% and 66%, Fisher’s *p* = 0.0219). The Random Forest ensemble also exhibited more uniform class-specific performance, with F1-scores of 0.77, 0.74, and 0.77 for HC, IPD, and DIP, respectively. Accordingly, the Random Forest balanced under-sampling ensemble was used for the primary multiclass results reported in Section 3.4.3 and Figure 9. Its confusion-matrix results correspond to 65/82 correctly classified HC participants, 60/82 IPD participants, and 62/82 DIP participants.

## Appendix C

### Appendix C.1. HHT Decomposition and Physiological Frequency Partitioning

The five retained IMFs exhibited systematically ordered empirical frequency distributions, spanning lower-frequency phonatory components to higher-frequency harmonic, formant, and perturbation components. Table A7 summarizes the empirical mean frequencies and 5th–95th percentile ranges for each IMF across the five vowels. These frequency distributions support the use of five IMFs as a consistent decomposition depth.

**Table A7:** Frequency Ranges per Vowel and IMF.

Vowel	IMF Component	Mean Frequency (Hz)	5th–95th Percentile Range (Hz)
/a/	IMF 0	3597.5	1292.7–5253.8
	IMF 1	1631.8	692.8–2992.5
	IMF 2	789.0	397.7–1315.5
	IMF 3	379.1	180.4–642.2
	IMF 4	198.1	110.8–276.4
/e/	IMF 0	3408.4	1299.3–5203.8
	IMF 1	1842.1	770.6–2943.8
	IMF 2	715.6	283.3–1404.5
	IMF 3	311.8	166.2–485.3
	IMF 4	153.4	79.0–237.1
/i/	IMF 0	3535.5	1429.0–5242.9
	IMF 1	1951.6	913.7–3063.7
	IMF 2	933.8	293.1–1731.9
	IMF 3	339.6	182.5–477.9
	IMF 4	155.0	82.7–202.5
/o/	IMF 0	3732.4	1474.9–5296.1
	IMF 1	1698.1	728.0–2940.2
	IMF 2	817.9	335.3–1394.6
	IMF 3	363.5	167.1–537.6
	IMF 4	189.3	91.0–268.1

### Appendix C.2. Reccurrence Quantification Analysis

#### Appendix C.2.1. Methodological Rationale for the Baseline Threshold (*ϵ* = 0.10)

Recurrence Quantification Analysis (RQA) quantifies the structural density, predictability, and laminar states of dynamical trajectories reconstructed in phase space. The selection of the recurrence threshold *ϵ* is critical: an excessively small threshold causes sparse matrix starvation (*RR* → 0), obscuring deterministic diagonal structures and capturing stochastic background noise, whereas an overly large threshold induces artificial saturation (*RR* → 1), creating ceiling effects where noise is misclassified as deterministic recurrence [35,62].

In our acoustic framework, the baseline threshold was fixed at *ϵ* = 0.10 based on two physical and acoustic principles:

1. Geometric Scale of Normalized State Space: Prior to phase-space embedding, all stable vocal time series undergo dynamic silence trimming (30 dB), removal of the initial 200 ms onset transient, and peak amplitude normalization to [−, 1.01.0]. In an *m*-dimensional phase space evaluated under the Chebyshev norm (*L*_∞_ distance metric, *d*_∞_(*y_i_*,*y_j_*) = *max_k_*∣*y_i_*_,*k*_ − *y_j_*_,*k*_∣), the maximum theoretical distance between any two state vectors within the attractor is bounded by *maxd*_∞_ ≈ 2.0. Consequently, setting *ϵ* = 0.10 corresponds to exactly 5% of the maximum attractor diameter (0.05 ⋅ *D*_max_, or 10% of the single-sided unit amplitude).
2. Canonical Recurrence Rate Bounds: In the established recurrence plot literature [35,62], an optimal threshold should yield recurrence rates within the canonical range of 5% to 30%. As shown in Figure A2, at *ϵ* = 0.10, the empirical Recurrence Rate across our sustained phonations averaged 16.4% in patients with DIP (ranging from 14.5% to 20.1% across vowels) and 23.4% in healthy controls (ranging from 18.0% to 34.9% across vowels). This dynamic range provides the optimal contrast to capture both diagonal lines (Determinism, DET) and vertical structures (Laminarity, LAM) without boundary distortion or phase-space starvation.

#### Appendix C.2.2. Threshold Sensitivity Analysis

To confirm that the observed differences in vocal recurrence dynamics between patients with DIP and healthy controls were invariant to the specific choice of threshold, a systematic sensitivity analysis was conducted across a three-fold threshold span (*ϵ* = 0.05, 0.08, 0.10, 0.12, 0.15) across all five cardinal vowels (/a/, /e/, /i/, /o/, /u/).

As detailed in Table A8 and illustrated in Figure A2:

- Invariance of Determinism (DET): Across the entire threshold spectrum (*ϵ* ∈ [0.05,0.15]), Determinism was significantly reduced in DIP patients compared to healthy controls (*p* < 0.05 across 23 of the 25 evaluated vowel–threshold conditions). The pathological effect size remained exceptionally robust across all cardinal vowels: vowel /a/ maintained a large effect size (*d* = −0.885 to −0.935, all *p* ≤ 0.0001), vowel /o/ ranged from *d* = −0.532 to −1.017 (all *p* ≤ 0.0188), vowel /e/ ranged from *d* = −0.578 to −0.680 (all *p* ≤ 0.0139), vowel /u/ ranged from *d* = −0.459 to −0.938 (all *p* ≤ 0.0723; *p* < 0.025 for *ϵ* ≤ 0.12), and vowel /i/ ranged from *d* = −0.397 to −0.461 (all *p* ≤ 0.0971; *p* < 0.05 for *ϵ* ≤ 0.10).
- Invariance of Laminarity (LAM): Laminar trapped states were consistently lower in DIP across all fixed thresholds in open and mid vowels, with Cohen’s *d* remaining steady across thresholds for vowel /a/ (*d* = −0.821 to −0.906, all *p* ≤ 0.0002), vowel /o/ (*d* = −0.509 to −0.580, all *p* ≤ 0.0319), vowel /e/ (*d* = −0.502 to −0.552, all *p* ≤ 0.0284), and vowel /i/ (*d* = −0.440 to −0.504, all *p* ≤ 0.0433), reflecting persistent disruption of vocal-fold contact stability across neighborhood scales.
- Suppression of Recurrence Rate (RR): Recurrence density was consistently suppressed in DIP across all fixed thresholds, reaching robust statistical significance in open and mid vowels: vowel /a/ (*d* = −0.817 to −0.966, all *p* < 0.0001), vowel /e/ (*d* = −0.454 to −0.605, all *p* ≤ 0.0447), and vowel /o/ (*d* = −0.371 to −0.497, all *p* ≤ 0.0491).

These findings demonstrate that the reduction in vocal recurrence, loss of deterministic fold trajectory, and disruption of laminar stability in DIP are intrinsic pathophysiological characteristics of laryngeal rigidity and tremor, rather than artifacts of a specific mathematical threshold.

**Table A8:** Sensitivity analysis of RQA parameters (DET, RR, LAM) across varying recurrence thresholds (*ϵ* ∈ [0.05, 0.15]) for all five cardinal vowels in DIP (*N* = 82) and healthy controls (*N* = 30). All statistical comparisons were computed at the independent subject level using two-sided Mann–Whitney *U* tests. Effect size *d* indicates Cohen’s *d* calculated with pooled sample standard deviation (ddof = 1); negative values denote reductions in DIP relative to controls.

Vowel	*ϵ*	DET Cohen’s d	DET *p*	RR Cohen’s d	RR *p*	LAM Cohen’s d	LAM *p*
/a/	0.05	−0.885	0.0001	−0.817	0	−0.906	0.0001
/a/	0.08	−0.892	0.0001	−0.914	0.0001	−0.871	0.0001
/a/	0.10	−0.898	0.0001	−0.966	0.0001	−0.821	0.0002
/a/	0.12	−0.928	0	−0.947	0.0001	−0.844	0.0001
/a/	0.15	−0.935	0	−0.935	0.0001	−0.837	0.0001
/e/	0.05	−0.68	0.0079	−0.605	0.0155	−0.543	0.0284
/e/	0.08	−0.664	0.0111	−0.559	0.0217	−0.536	0.0232
/e/	0.10	−0.656	0.0038	−0.52	0.0209	−0.552	0.0077
/e/	0.12	−0.615	0.0139	−0.489	0.0314	−0.519	0.0209
/e/	0.15	−0.578	0.0129	−0.454	0.0447	−0.502	0.0232
/i/	0.05	−0.442	0.0427	−0.332	0.0703	−0.504	0.028
/i/	0.08	−0.461	0.0352	−0.338	0.0703	−0.494	0.0347
/i/	0.10	−0.459	0.044	−0.335	0.0894	−0.488	0.0376
/i/	0.12	−0.437	0.0607	−0.315	0.0971	−0.465	0.0347
/i/	0.15	−0.397	0.0971	−0.291	0.135	−0.44	0.0433
/o/	0.05	−1.017	0	−0.497	0.0087	−0.562	0.0152
/o/	0.08	−0.833	0	−0.431	0.0248	−0.57	0.0185
/o/	0.10	−0.744	0.0002	−0.429	0.0325	−0.58	0.0192
/o/	0.12	−0.638	0.0028	−0.388	0.0433	−0.534	0.0261
/o/	0.15	−0.532	0.0188	−0.371	0.0491	−0.509	0.0319
/u/	0.05	−0.938	0.0001	−0.48	0.2305	−0.353	0.1667
/u/	0.08	−0.764	0.0024	−0.373	0.3949	−0.311	0.1728
/u/	0.10	−0.663	0.0082	−0.269	0.6106	−0.276	0.3101
/u/	0.12	−0.579	0.0248	−0.246	0.5477	−0.29	0.2131
/u/	0.15	−0.459	0.0723	−0.177	0.6669	−0.269	0.3038

## Appendix D. Machine Learning Permutation Test

To rule out the possibility that classification was driven by arbitrary dataset partitions or environmental artifacts, we conducted non-parametric Monte Carlo permutation tests by randomly shuffling diagnostic labels across 1000 iterations while holding the feature matrices constant. The empirical null distributions for both cohorts centered tightly around random chance (mean permuted AUC ≈ 0.500, 95th percentile ≤0.596). None of the 1000 permutations surpassed the actual model performances, yielding empirical *p*-values of *p*_perm_ < 0.001 across all evaluations.

**Table A9:** Permutation Tests of RF and XGBoost Model Classifications.

Cohort & Model	Metric	Observed (*M*^(obs)^)	95% Bootstrap CI	Permuted Null Mean (*μ*_null_)	Permuted 95th Percentile	*p*_perm_
DIP vs. HC (RF)	ROC-AUC	0.850	[0.759, 0.922]	0.498	0.596	<0.001
	Accuracy	85.7%	[78.6%, 92.0%]	66.5%	73.2%	<0.001
DIP vs. HC (XGBoost)	ROC-AUC	0.800	[0.697, 0.890]	0.498	0.596	<0.001
	Accuracy	78.6%	[70.5%, 85.7%]	63.1%	71.4%	<0.001
IPD vs. HC (RF)	ROC-AUC	0.965	[0.957, 0.974]	0.501	0.528	<0.001
	Accuracy	89.7%	[88.0%, 91.4%]	50.0%	52.4%	<0.001
IPD vs. HC (XGBoost)	ROC-AUC	0.981	[0.975, 0.987]	0.501	0.529	<0.001
	Accuracy	92.9%	[91.5%, 94.4%]	50.0%	52.6%	<0.001
